# ZEB1 Links p63 and p73 in a Novel Neuronal Survival Pathway Rapidly Induced in Response to Cortical Ischemia

**DOI:** 10.1371/journal.pone.0004373

**Published:** 2009-02-04

**Authors:** Thai Bui, Judith Sequeira, Tong Chun Wen, Augusto Sola, Yujiro Higashi, Hisato Kondoh, Tom Genetta

**Affiliations:** 1 Department of Neurology, Emory University School of Medicine, Atlanta, Georgia, United States of America; 2 Department of Pathology and Laboratory Medicine, Emory University School of Medicine, Atlanta, Georgia, United States of America; 3 MidAtlantic Neonatology Assoc., Mooristown, New Jersey, United States of America; 4 Perinatology, Institute for Developmental Research, Kasugai, Aichi, Japan; 5 Graduate School of Frontier Biosciences, Osaka University, Osaka, Japan; Istituto Dermopatico dell'Immacolata, Italy

## Abstract

**Background:**

Acute hypoxic/ischemic insults to the forebrain, often resulting in significant cellular loss of the cortical parenchyma, are a major cause of debilitating injury in the industrialized world. A clearer understanding of the pro-death/pro-survival signaling pathways and their downstream targets is critical to the development of therapeutic interventions to mitigate permanent neurological damage.

**Methodology/Principal Findings:**

We demonstrate here that the transcriptional repressor ZEB1, thought to be involved in regulating the timing and spatial boundaries of basic-Helix-Loop-Helix transactivator-mediated neurogenic determination/differentiation programs, functions to link a pro-survival transcriptional cascade rapidly induced in cortical neurons in response to experimentally induced ischemia. Employing histological, tissue culture, and molecular biological read-outs, we show that this novel pro-survival response, initiated through the rapid induction of p63, is mediated ultimately by the transcriptional repression of a pro-apoptotic isoform of p73 by ZEB1. We show further that this phylogenetically conserved pathway is induced as well in the human cortex subjected to episodes of clinically relevant stroke.

**Conclusions/Significance:**

The data presented here provide the first evidence that ZEB1 induction is part of a protective response by neurons to ischemia. The stroke-induced increase in ZEB1 mRNA and protein levels in cortical neurons is both developmentally and phylogenetically conserved and may therefore be part of a fundamental cellular response to this insult. Beyond the context of stroke, the finding that ZEB1 is regulated by a member of the p53 family has implications for cell survival in other tissue and cellular environments subjected to ischemia, such as the myocardium and, in particular, tumor masses.

## Introduction

Hypoxic/Ischemic (H-I) insult to the CNS is an important cause of neurological morbidity, with severe cases resulting in life-long deficits [1], [2]. Stroke is a leading cause of death in adults, and increasingly sophisticated imaging technologies reveal that the incidence of stroke in infants (particularly premature babies, 1 in greater than 4000 births) now rivals that seen in the over-70 population [3], [4]. Whatever the developmental time frame, H-I insult to the brain results in a net loss of both neuronal and glial cells through both apoptotic and necrotic mechanisms [for reviews], [see 5 and 6]. Although developmental distinctions clearly exist in the molecular etiology of H-I lesions, our understanding of these molecular events is woefully incomplete [7], [8].

The development of the cerebral cortex is an extraordinarily dynamic process, involving the migration, proliferation and turnover of vast numbers of cells [9]. A critical part of this process is the regulated elimination of excess neurons and glia through programmed cell death [10]. Histological studies in embryonic mice reveal a tremendous amount of cellular death in the developing cortex, in both neural progenitor and newly born neuronal populations, peaking at roughly 70% of the total cell number around embryonic day 14, and declining thereafter [11]. Work from a number of labs has implicated the involvement of transcriptionally active isoforms of p53 and p63 in this process [12], [13] and TAp63 has additionally been shown to be required for growth factor-deprivation-mediated apoptosis of developing sympathetic neurons [14]. The deltaN isoforms of p63 and p73 (functioning as dominant negative inhibitors of their transcriptionally active counterparts) are, as well, required for post-natal survival of both sympathetic and CNS neurons [15], [16], [17]. Whatever the mechanistic basis, proper development requires that cells of the immature cortex be biochemically and genetically primed to receive and readily respond to pro-death signaling, rendering them particularly vulnerable to H-I episodes.

ZEB1/delta EF1, a phylogenetically conserved DNA-binding transcriptional repressor, has been implicated in the regulation of a number of basic-Helix-Loop-Helix (bHLH; e.g. MASH1) target genes [18], [19], [20]. Such a role is not, however, supported by the phenotype of the ZEB1 KO mouse, in which bHLH-triggered differentiation programs appear unaltered. Rather, at birth, ZEB1 knockout mice are about one-third to one-half proportionately smaller in size than their heterozygous or wild-type littermates [21], consistent with it playing a role in cellular survival (and/or in regulation of the cell cycle). Here, we present data that strongly supports such a role for ZEB1 in neuronal survival in the context of ischemic insult in the CNS, by serving as a critical link in a transcriptional cascade involving pro-apoptotic isoforms of p63 and p73. Specifically, we demonstrate that ZEB1 protein expression levels in cortical neurons in vivo are highly induced early in response to the experimental administration of permanent unilateral stroke in both the developing and mature brain. Similar levels of induction of human ZEB1 protein are seen in cortical samples derived from pathology specimens of stroke victims. Over-expressed ZEB1 is protective in primary cortical neurons subjected to oxygen-glucose deprivation (OGD), while, conversely, primary cultures of cortical neurons from ZEB KO mice are over twice as vulnerable to this insult. The ischemic increase in ZEB1 results, in part, through transcriptional induction by p63, itself induced early in response to stroke. We show further that the neuro-protective effects mediated by this increase in ZEB1 protein are the consequence, at least in part, of its binding to and repressing the TAp73 gene. This is the first demonstration, through the elucidation of this novel transcriptional cascade, of a functional role for ZEB1 in cell survival, and in neuronal protection in particular.

## Results

### ZEB1 protein is rapidly induced in cortical neurons by ischemia

**Figure 1:**
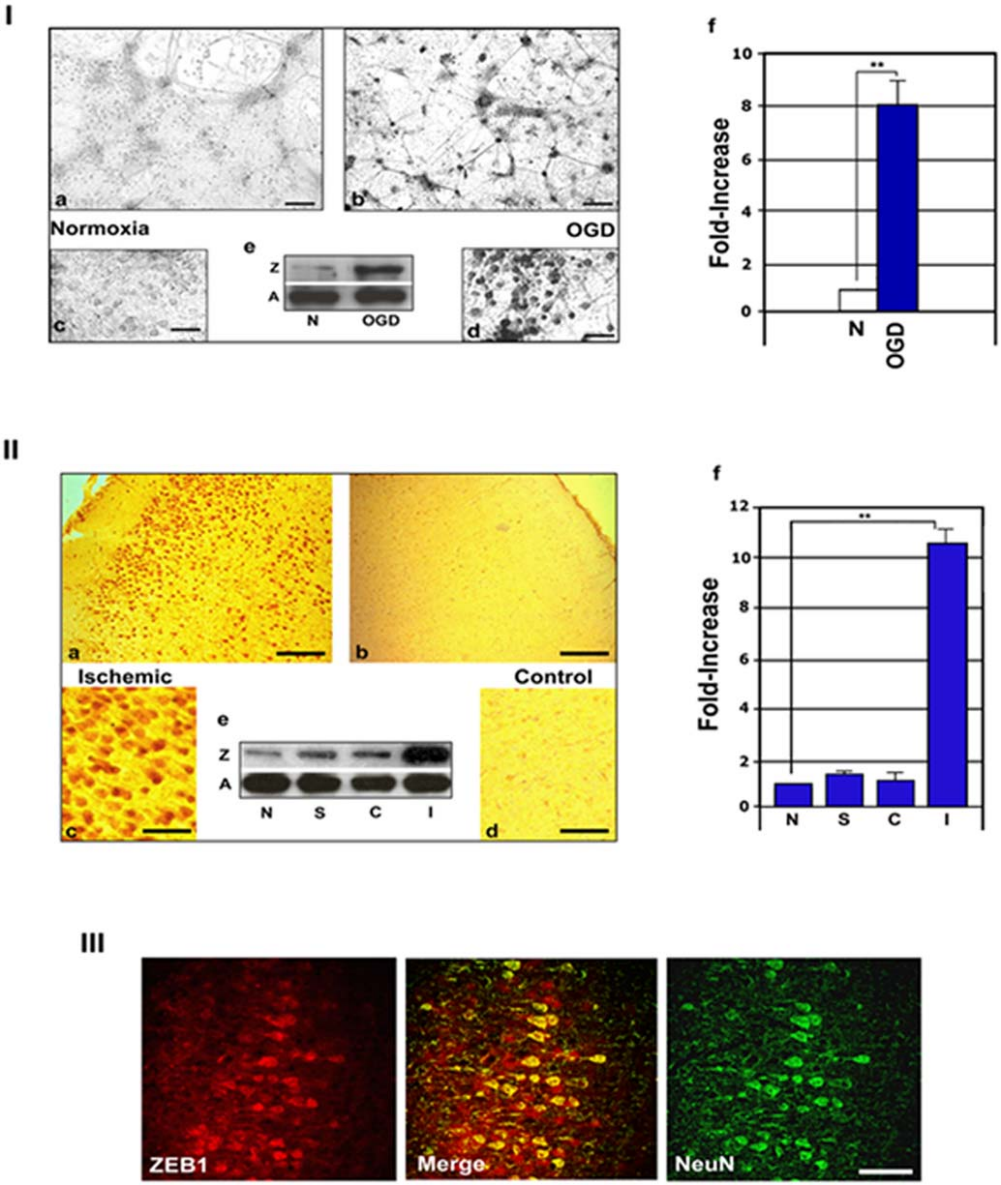
Ischemia-mediated induction of ZEB1 protein in cortical neurons. Specificity of the ZEB1 antibody used in these studies is demonstrated in Figure S1; Panel I. ZEB1 protein levels increase 8-fold in primary cultures of cortical neurons exposed to OGD. Photomicrographs of E16.5 primary cortical neuronal cultures exposed to normoxia (a,c), or OGD (b,d) for 90 minutes and immuno-stained for ZEB1 protein; (e), representative western analysis of total protein isolates, Z = ZEB1, A = β-actin, N = normoxia; f, densitometry of the bands from e, normalized to β-actin; four different replicate samples of cells (isolated on different days) were scored and the average+/−the S.E.M. is shown; Panel II. ZEB1 protein levels increase over 10-fold in the P7 rat ischemic cortex. Representative photomicrographs of immunostained coronal sections comparing the ischemic (a,c) and the contra-lateral (b,d) sides of the same sections, harvested 3 hours following administration of unilateral permanent FCI; the increase is largely confined to the nucleus of cells located mainly in the inner and outer pyramidal cell layers of the cortex; (e), representative western analysis of total cortical protein isolates; f, densitometry of the bands from (e), normalized to β-actin; N = normal brain, S = sham, C = contra-lateral, I = ischemic; Graph depicts the average of at least three separate experiments, and the average+/−the S.E.M. is shown; Panel III. Cells up-regulating ZEB1 in the ischemic cortex co-localize with the neuronal marker NeuN. Representative staining of 8 µm coronal sections from P7 rat pup brains harvested 12 hrs. post-FCI. Scale bars: panel I: a,b, 250 µm; c,d, 100 µm; panel II: a,b, 1 mm; c,d, 50 µm; panel III: 50 µm. ** = P<0.01, by Student's t-test.

A recent report demonstrating that a shift in intracellular redox levels to a net reducing environment (for example, as a result of hypoxia) dramatically increased the functional activity of a ZEB1 co-repressor, C-terminal binding protein 1 [CtBP1; 22], prompted us to ask whether ZEB1 may itself be subject to regulation by O_2_ levels in the brain. As a first approach, we subjected primary cultures of neurons isolated from cortexes of E16.5-E17.5 rats to OGD for various times, and immuno-stained them for ZEB1 expression. The proportion of neuronal cells in these cultures was assessed by immuno-staining a representative culture with appropriate anti-neuronal (anti-NeuN or MAP2) or anti-glial (GFAP) antibodies, and was consistently greater than 95% neuronal (not shown). As seen in **Figure 1, panel I**, a 2 hr. exposure to OGD (<0.5% O_2_), without subsequent re-exposure to normoxia, induced a clear increase in the levels of ZEB1 protein in these neurons compared to normoxic controls (**panels b and d vs. c and e**). Western blots on protein isolates from cultures treated in parallel revealed an average increase in ZEB1 protein levels to be just under 8-fold under these conditions (inset, **Figure 1, panel I, e**; a graphic representation of this increase is depicted in **panel I,f**).

### ZEB1 is induced in cortical neurons in vivo in response to experimentally-induced ischemic stroke

We then asked whether ZEB1 is similarly regulated in vivo as a consequence of experimentally-induced stroke. Permanent focal cerebral ischemia (FCI) was induced in P7 rat pups (the approximate developmental analogue of the human brain near term; for details, see [23]), via suture embolism of the mid-cerebral artery, and in adult rats by electro-coagulation of that same artery, and brains were harvested and processed for immuno-histochemistry at various time points following the injury. Beginning about 90 minutes post-op, we detected an average 10-fold increase (normalized to β-actin levels: see **Figure 1, panel II, f**) in ZEB1 protein levels in the cortex of the ischemic side (in what appear to be the internal and external pyramidal cell layers of the cortex, **Figure 1, panel II, a and c**). The response in the adult rat brain was over twice that seen in the neonate, with the area of infarct more closely confined to the region in the cortex proximal to the occluded mid-cerebral artery (data not shown). At either stage of development, in this model of permanent MCAO, increased ZEB1 protein expression in the ischemic cortex persisted for at least 72 hrs., co-incident with the radiating penumbra in the growing lesion (data not shown). Verification that ZEB1-positive cells in the ischemic cortex are neurons (likely pyramidal cells, based on their morphology) was obtained by double-immunofluorescence staining frozen sections using antibodies specific for ZEB1 and the neuron-specific marker NeuN (**Figure 1, panel III**).

### Primary neurons from ZEB1 knock-out mice are significantly more vulnerable to OGD

**Figure 2:**
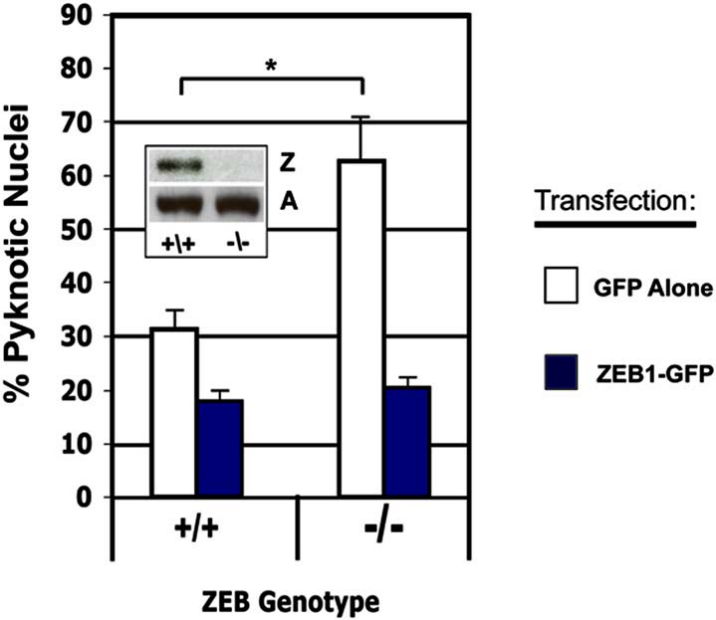
Primary cortical cultures derived from ZEB1 KO or wt E16.5 embryos were transfected with either a GFP expression vector (control, White Bars), or a plasmid expressing a full-length ZEB1 cDNA fused to GFP (Blue Bars). Sixteen hours later, cells from both groups were subjected to OGD for 6 hrs, fixed under hypoxia, the nuclei were Hoechst-stained and then scored (in a blinded fashion) for presence of either a normal vs pyknotic/mis-shapen/condensed morphology. Over twice as many pyknotic nuclei were scored in ZEB KO cells as compared to their wt counterparts. Insert shows a representative western blot verifying the absence of ZEB1 protein in neurons derived from the KO animal. Lower bands are the loading control β-actin. Over-expressing ZEB1 cDNA conferred a similar increase in resistance above wt (Blue Bars) on neurons of either genotype. Four different replicate samples of cells (isolated on different days) were scored and the average+/−the S.E.M. is shown; Z, ZEB1; A. β-actin; * = P<0.05, by Student's t-test.

Mice in which the ZEB1 gene has been deleted are proportionally one-half to two-thirds the size of their wild-type littermates [21]. While not dispositive, such a phenotype is consistent with this transcriptional repressor participating in the regulation of genes involved in promoting cell death. Further support for such a role comes from the recent finding that a ZEB1 co-repressor, CtBP1, can itself repress a subset of pro-apoptotic genes [24, and see discussion]. If ZEB1 functions, at least in part, as an early defense mechanism initiated in cortical neurons to promote their survival following ischemia, ZEB1−/−neurons in culture might be more vulnerable to the approximate tissue culture equivalent, oxygen-glucose deprivation (OGD). To test this, we isolated primary cortical cultures isolated from ZEB1−/−mouse E16.5 mouse embryos (as well as from their wt littermates) and subjected them to this insult. After 6 hrs in OGD, we detected over twice as many pyknotic/condensed nuclei in ZEB1−/−neurons compared with wt (**Figure 2, white bars**). Over-expressing ZEB1 via transfection in these cells rescues their resistance to OGD to levels similar to transfected wt cells (**Figure 2, blue bars**).

Although the specific mechanism – apoptosis, necrosis, autophagy, etc. – depends on many factors, including region of the brain affected, developmental stage, and the type, degree and duration of insult, the ultimate pathological consequence of severe, sustained ischemic insult is cell death [6], [7], [8]. To address the potential role of ZEB1 in this process, we asked whether the ischemia-induced neuronal up-regulation of this protein co-localized with the induction of specific, well-characterized, cell-death/survival-related markers. Double-immunofluorescence staining revealed that, in the ischemic cortex 12 hrs-post insult, cells staining positive for either active Caspase 3 (**Figure S2, top**), or the BH3-only, Bcl-2 family member PUMA (**Figure S2, bottom**), both well characterized markers for cells undergoing apoptosis [25], [26], were virtually all mutually exclusive from ZEB1-positive cells.

### Over-expressing ZEB1 protein in primary cortical cultures protects them from specific pro-death insults

**Figure 3:**
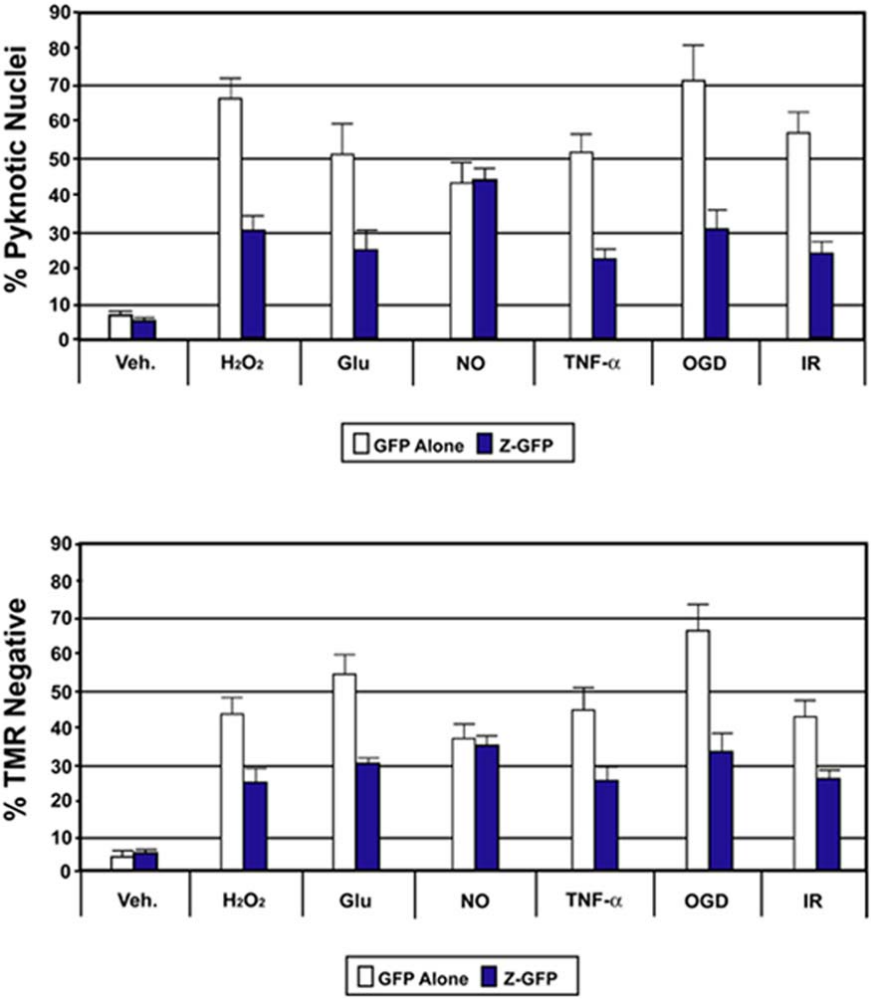
Over-expressed ZEB1 protects primary cortical neurons from a subset of pro-death insults. E16.5 primary cortical neurons transfected with GFP or ZEB1 fused in frame to GFP (for a 30 hr period) were subjected to the following pro-death agents for the indicated times: Veh., DMSO; H2O2, hydrogen peroxide (6 hrs); Glu, glutamic acid (18 hrs); NO, nitric oxide (20 hrs); TNF-a, tumor necrosis factor alpha (18 hrs); OGD, oxygen-glucose deprivation (12 hrs); IR, ionizing radiation (6 hrs). At indicated time points, cells were fixed, processed and their ability to resist cellular degradation/death was assessed using two distinct markers of cellular degradation or death. Representative fluorescence images from these analyses are shown in Figures S3 (nuclear morphology) and S4 (mitochondrial membrane integrity). Top graph: in every case except for NO, at a given concentration/level of the indicated treatment, and at the time points examined, on average, less than half as many nuclei in neurons over-expressing ZEB1 showed a shrunken/pyknotic/condensed morphology compared with their GFP-expressing counter-parts. Bottom graph: in every case except for nitric oxide, at a given concentration/level of the indicated treatment, and at the time points examined, on average, over twice as many ZEB1-over-expressing neurons maintained intact mitochondrial membranes compared with their GFP-expressing counterparts. Results shown above are averaged from at least three separate experiments (cultures isolated on different days)+/−the S.E.M. ** = P<0.05 by ANOVA.

To further test whether ZEB1 protein induction is part of a neuro-protective mechanism, we scored the relative health of ZEB1-transfected neurons subjected to a battery of pro-death insults using two independent histological methods (chosen, in part, because of their extensive association with stroke-mediated neuropathology and neuronal cell death): either mitochondrial- (loss of membrane integrity) or nuclear- (morphological integrity) based. Following transfection of either a full-length ZEB1-GFP fusion protein or with GFP alone, cells were challenged with a range of individual insults, including OGD, and then scored at specific time points for their ability to resist cellular degradation/death (representative fluorescence images from these analyses are shown in **Figures S3** [nuclear morphology] and **S4** [mitochondrial membrane integrity]). As shown in **Figure 3**, compared with controls, neurons over-expressing ZEB1 (blue bars) displayed a significantly greater survival capacity, by either histological measure, in the face of H_2_O_2_, glutamate, TNF-α, ODG, and ionizing radiation. The lone exception, nitric oxide (NO), has been studied extensively in both normal and patho-physiological contexts in the CNS, and, depending on many factors, can mediate either neuroprotective or neurotoxic effects [45], [46]. The NO result is especially interesting given that over-stimulation of NMDA receptors via excitotoxic insult has been shown in cortical neurons to increase (via calcium-mediated activation of neuronal nitric oxide synthase (nNOS)) intracellular levels of NO-derived reactive nitrogen species, which potentially can play a role in subsequent cellular damage and death [45]–[49]. Whether the failure of ZEB1 to mitigate or delay the NO-mediated toxicity seen in the context of the relatively pure primary neuronal cultures employed here can offer any mechanistic insight into ZEB1's neuroprotective effects will depend on a number of factors (see discussion).

We then followed a time course of ZEB1-mediated protection of neurons in culture from the introduction of double-stranded DNA breaks – a classic hallmark cellular injury/death – resulting from oxygen-glucose deprivation, one of the critical injury-inducing consequences of in vivo ischemic episodes. Significant protection (greater than 2-fold) was achieved at least 6 hrs following the administration of this insult, as revealed through TUNEL staining (**Figure S5**).

### ZEB1 up-regulation is mediated transcriptionally, in part, by ischemia-induced p63

Quantitative real-time-PCR (qRT-PCR) indicated that approximately 70% of the increase in ZEB1 protein levels seen in OGD-treated primary neurons could be ascribed to an induction in the steady-state levels of ZEB1 message (**Figure S6**). A computer-based search [27] for transcription factor-binding sites within the immediate-upstream regulatory region (proximal promoter) of the ZEB1 gene revealed the presence of a phylogenetically-conserved p53-family binding site. Given the well-established roles that p63 and p73 play in mediating cell death/survival in both CNS and PNS neurons [16], [17; for review], [see 14], we asked whether a member of the p53 family might, through direct regulation of the ZEB1 gene, participate in the determination of the survival outcome of cortical neurons subjected to ischemic insult.

**Figure 4:**
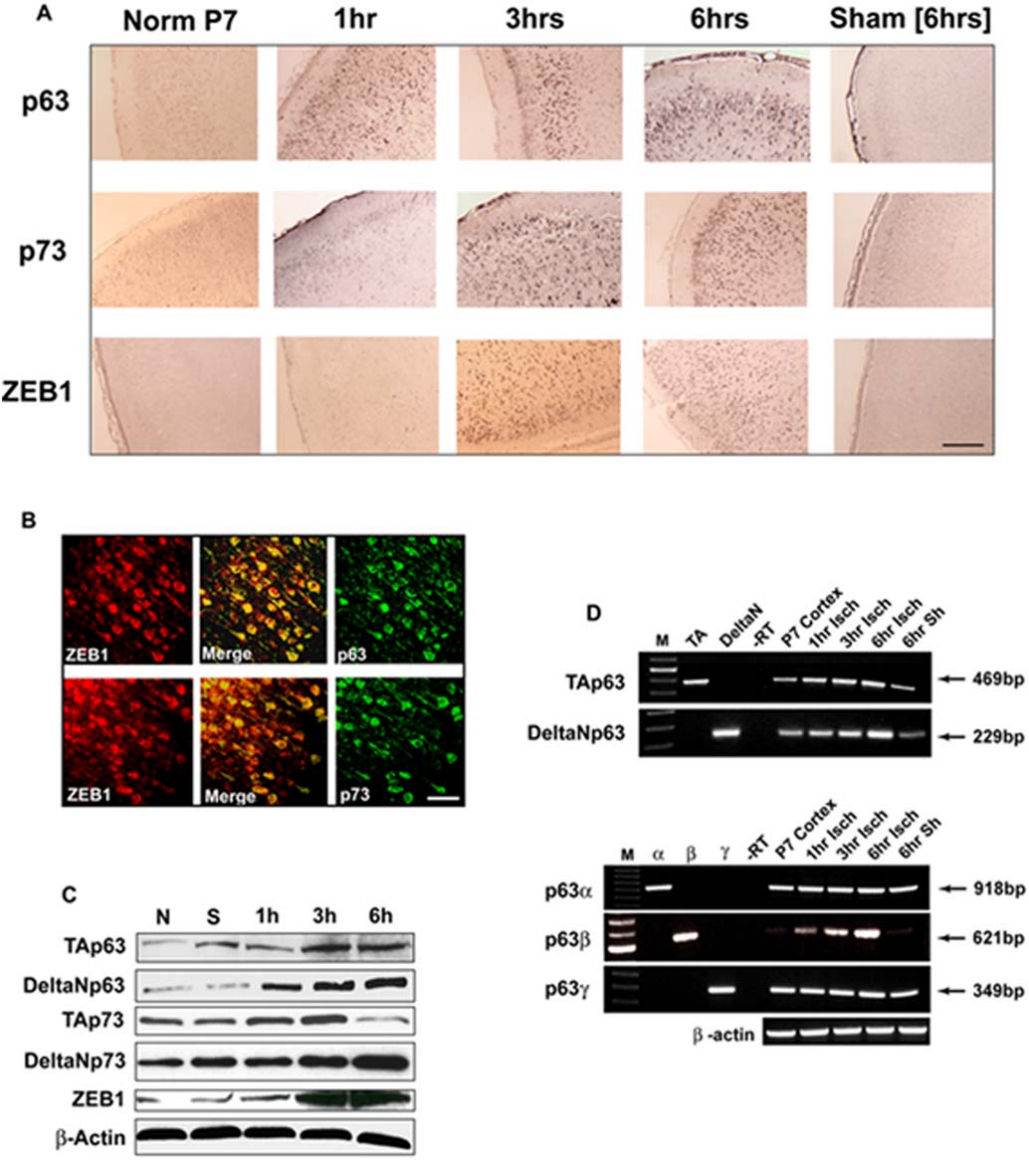
p63 is rapidly induced in response to ischemia. (A) Expression profile of p63, p73 and ZEB1 protein induction in the ischemic cortex of P7 rat pups subjected to FCI. Brains were harvested and processed for immunostaining at the indicated time points post-FCI. Colorimetric staining reveals that p63 is induced the earliest, at least one hr post-FCI. Scale bar = 500 µm. (B) Both p63 and p73 induction co-localize with that of ZEB1. Double-immuno-fluorescence staining indicates that all three of these proteins co-localize to neurons in the ischemic cortex. In all cases, staining was carried out at least three times and typical results are shown. Scale bars, a., 500 µm; b., 50 µm. (C) Western analysis of the induction of p63 and p73 isoforms compared to ZEB1. Total cellular protein was isolated from normal (N), sham operated (S – 6 hr time point), or ischemic cortexes at the indicated time points following administration of FCI. All show some degree of up-regulation in this immediate time frame, except for TAp73, which after an initial rise, declines between 3 and 6 hrs post FCI. b-actin is included as a loading control. Shown is a typical result from separate analysis of three different experiments. (D) RT-PCR analysis of temporal expression of p63 isoforms in the cortex immediately following FCI. Primer pairs (see Materials and Methods) were designed to specifically amplify the isoform classes indicated but could not distinguish whether a particular 3′ splicing variant (α β or γ) harbored the TA or the DeltaN 5′ end. While p63 TA, alpha and gamma both showed a modest increase in the six-hr period immediately following FCI, the beta isoform showed a dramatic induction. Positive controls are shown in the first two lanes in the top panel and in the first three lanes in the bottom panel. Sizes of PCR products are indicated on the right.

Similar to previously published results employing an adult rat stroke model [28], an increase in p53 protein itself was not observed histologically in the ischemic cortex until at least 6 hrs post-insult (data not shown). p73 protein expression increased slightly one hr post-insult, and continued to increase, reaching a plateau at around 3 hrs post-insult. p63 protein was induced even earlier, within at least one hr following the ischemic injury (**Figure 4A**). Double immuno-fluorescence staining showed that the induction of both p63 and p73 protein co-localized with that of ZEB1 protein in cortical neurons (**Figure 4B**). Western analysis of total cellular protein lysates from ischemic vs normal or sham operated P7 rat cortexes showed that the TAp63, ΔNp63, and ΔNp73 protein isoforms all increased over the 6 hr time period examined, with the ΔNp63 isoform increasing the most rapidly, within one hour post-insult (**Figure 4C**). Interestingly, the pro-apoptotic TAp73 isoform [29], following an initial increase, *decreased* between 3 and 6 hrs post-insult (**Figure 4C**), co-incident with the steadily increasing levels of ZEB1, ΔNp63, TAp63 and ΔNp73.

These temporal protein induction profiles suggested that p63 might participate in the transcriptional up-regulation of the ZEB1 gene through the DNA binding site described above. To address this question, we analyzed total RNA isolates from normal, sham-operated and ischemic cortexes at 1, 3 and 6 hrs post-insult for the presence of various p63 mRNA isoforms via RT-PCR. While the ΔN, 63α and p63γ isoforms all increased slightly over the 6 hr period, the p63β isoform rose dramatically (**Figure 4D**).

**Figure 5:**
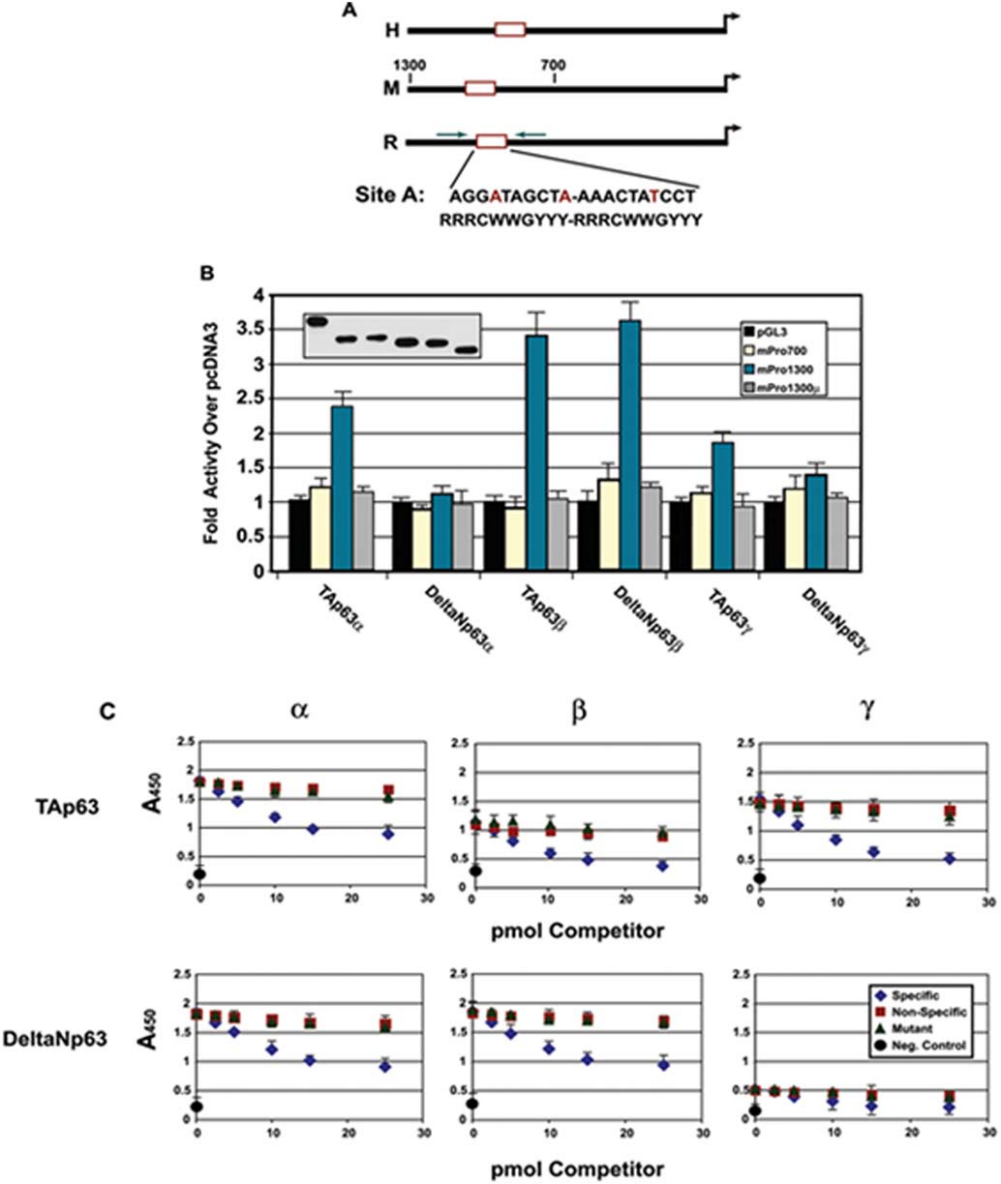
p63 binds and transactivates through a phylogenetically conserved binding site in the proximal promoter region of the ZEB1 gene in response to ischemia. (A) Schematic of the promoter regions of human (H) mouse (M) and rat (R) ZEB1 genes. The binding site in the rat gene is shown, with the bases differing from the p53 binding site consensus sequence shown in red. (B) Specific p63 isoforms can transactivate the ZEB1 promoter. The listed p63 isoform cDNAs were co-transfected into the p63-null cell line Saos-2 along with a luciferase reporter plasmid driven by the fragments (listed in the right panel insert) of the mouse ZEB1 promoter. The luciferase activity derived from the co-transfection of TAp63α, TAp63β and DeltaNp63β isoforms was dependent on the p53 binding site, the mutation of which abrogated this activity (gray bars); insert is a western blot derived from equal amounts of protein lysates from parallel transfected cultures using a pan-anti-p63 antibody (identity of bands follows the same order, left to right, as the transfection data). (C) Equimolar amounts of protein derived from in vitro translated p63 isoform cDNAs were used in a micro-titre-based DNA-binding assay employing oligonucleotides harboring the site shown in panel (A) (see Materials and Methods). With the exception of the DeltaNγ isoform, all bound specifically to the site in the rat promoter. Competitor oligos are identified in the panel insert.

**Figure 6:**
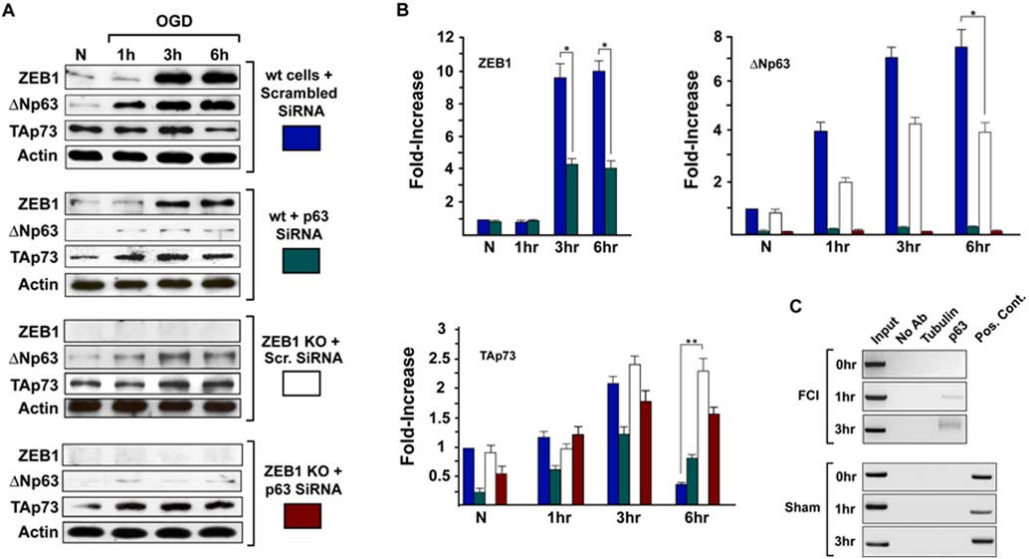
OGD-mediated ZEB1 protein induction depends significantly on p63. (A) Primary cortical cultures from either wt or ZEB1 KO mice were transduced with lentiviral constructs expressing either scrambled or p63 SiRNA (see Materials and Methods for sequences). Western blots of protein lysates prepared at indicated time points were carried out using antibodies shown at left. Densitometry of normalized (to β-actin) expression/induction profiles are depicted graphically in (B). Results are representative of 3 separate experiments, carried out on independent primary neuronal isolates (done on different days). At the 3 hr time point, SiRNA-mediated reduction in p63 protein levels reduces OGD-mediated ZEB1 protein induction an average 60% (ZEB1 graph). The reduction in p73 protein levels in wt neurons between 3 and 6 hrs following OGD (see Figure 4, panel C, and the top panel of westerns, labeled: “wt cells+scrambled SiRNA” in this figure) is virtually eliminated in the absence of ZEB1 (TAp73 graph). Interestingly, the dramatic increase in OGD-mediated ΔNp63 protein levels (seen in the top panel of westerns, labeled: “wt cells+scrambled SiRNA”) was reduced by half in a ZEB1 KO background (see text). (C) ChIP assay using the pan-anti-p63 antibody (recognizing all isoforms in the transfection analysis, Figure 5, panel B) shows that, in the cortex, within 1 hr of FCI, p63 binds to the site depicted in Figure 5, panel A in vivo. Primers used are depicted schematically (in green) in Figure 5, panel A. Tubulin, negative control ChIP using heterologous anti-Tubulin Ab; Pos. Cont., PCR product from post-IP supernatant harboring p63 binding sites (see Methods). Error bars: S.E.M. * = p<0.01; ** = p<0.001.

Transactivation analysis, coupled with DNA-binding assays revealed that TAp63α, TAp63β and ΔNp63β could both bind and transactivate the ZEB1 promoter through the above site (**Figure 5B and C**). To verify whether and to what extent p63 participates in the OGD-mediated increase in ZEB1 protein, we reduced endogenous p63 levels in primary cultures of mouse cortical neurons via lentiviral-mediated SiRNA [30] and measured ZEB1 protein induction in control vs OGD-treated cells. The results (**Figure 6A**, shown graphically in **6B**) demonstrate that, in this experimental context, ΔNp63 accounts for roughly 60% of ZEB1 protein induction (see also **Figure 4C and D**). Interestingly, in control experiments with (**Figure 6A**) or without (not shown) scrambled SiRNA transduced into primary cultures of ZEB−/−neurons (maintained in the presence of the pan-Caspase inhibitor z-VAD-fmk), not only was the reduction in TAp73 protein levels between 3 and 6 hrs of OGD treatment (as occurs in a wt ZEB1 background) virtually eliminated, but the OGD-mediated induction of ΔNp63 protein – about seven-fold in a wt ZEB1 background – was itself reduced by half (see discussion). Lastly, we demonstrated in vivo binding of p63 to the ZEB1 promoter (in the ischemic cortex vs. sham operated), employing a pan-p63 antibody (recognizing all isoforms) in a chromatin immunoprecipitation (ChIP) assay (**Figure 6C**).

### ZEB1 represses the pro-apoptotic p73 isoform in response to FCI

**Figure 7:**
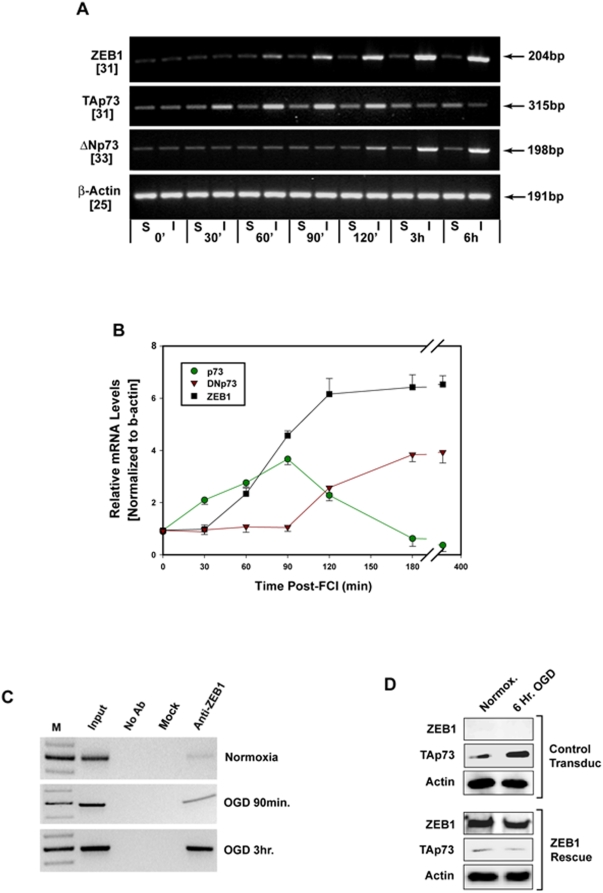
ZEB1 represses TAp73 in the cortex in response to ischemic insult. (A) Agarose gel of RT-PCR analysis of cDNA synthesized from total RNA isolated at the indicated time points from either Sham-operated (S) or FCI (I) P7 cortexes. The numbers in parentheses on the left (below the indicated PCR product) denote the number of PCR cycles used to achieve linear-range amplification of each target. Shown is a representative result from one of three independent experiments (each derived from a separate litter). To control for the presence of contaminating genomic DNA, a PCR-only reaction using identical conditions was carried out using these samples (not shown). (B) For each experiment, a numerical value for relative intensity was obtained by densitometry, and after normalization to the levels of β-actin (bottom row, panel a) Sham (background) values were subtracted from the corresponding Ischemic values. The average of these results from all three RT-PCR analyses are depicted graphically as the average value+/−the S.E.M. Two hours after the administration of FCI, steady-state levels of ZEB1 mRNA (black squares) are beginning to plateau, those of TAp73 (green circles) are declining, while ΔNp73 levels are increasing. (C) ChIP analysis showing increased ZEB1 protein binding to the previously characterized silencer region within intron 1 of the human p73 gene, beginning one hour after primary cortical neurons were subjected to OGD, and increasing significantly at three hours. Results shown are representative of three separate experiments. (D) Rescue of ZEB1-mediated repression of p73 protein levels. Primary cortical cultures isolated from ZEB1 knock-out late stage embryos were transduced with a lentivirus harboring either GFP (“Control Transduc”, top) or ZEB1 fused to GFP (“ZEB1 Rescue”, bottom). The top three panels in (D) were derived from a (representative) single western blot, and the bottom three panels from a different (representative) single western blot, each the product of one of three separate experiments (cultures isolated on different days). Restoration of ZEB1 re-established the OGD-mediated reduction in TAp73 protein (see Figure 4 Panel C, Figure 6, panel A).

Not only does TAp73 participate in the apoptotic elimination of excess neurons in the developing CNS and PNS [for review, see 14], but ZEB1 has been demonstrated to repress the p73 gene (by binding to a silencer region in the first intron of the human homologue of that gene) in an in-vitro model of neuronal differentiation [31]. These findings, together with the above data, compelled us to explore the regulatory link between ZEB1 and p73 in ischemic neurons by asking whether the protective effect of ZEB1 is mediated, at least in part, through its repressing the p73 gene. We addressed this by comparing mRNA expression profiles of ZEB1, TAp73α and ΔNp73 in the P7 cortex in the immediate post-ischemic (6 hr) time frame. Consistent with our analyses at the protein level (**Figures 4C** **and** **6A**), the steady-state levels of TAp73 mRNA begin to decline as ZEB1 mRNA levels are seen to rise (between 90 mins and 2 hrs post-insult; **Figure 7A**, summarized graphically in **Figure 7B**). Coincident with this, steady-state levels of the pro-survival ΔNp73 isoform [15], [16] mRNA levels begin to rise after about 2 hrs. Using OGD-treated vs. normoxic-treated primary cultures of human embryonic cortical neurons, a ChIP assay showed that ZEB1 does indeed bind to the previously characterized intron 1 silencer region of the human p73 gene in response to this insult (**Figure 7C**). To demonstrate this point further, we were able to rescue the OGD-mediated down-regulation of TAp73 protein, by re-introducing ZEB1, via lentiviral transduction, back into cortical neurons isolated from E16.5 ZEB1 knock-out mouse embryos (**Figure 7D**). Together with our data showing that p73 protein levels fail to decline in OGD-treated ZEB−/−neurons (**Figure 6A**), these results are consistent with ZEB1 playing a direct role in repressing the p73 gene, offering one mechanistic basis for it's protective effect.

### ZEB1 protein is up-regulated in the human brain in response to ischemic stroke

**Figure 8:**
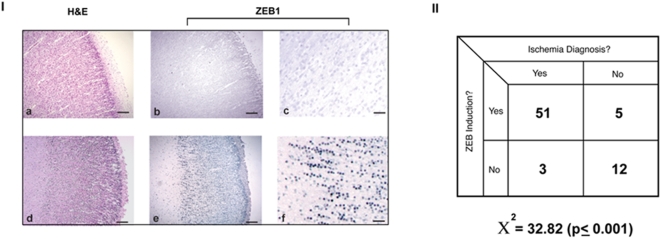
ZEB1 protein is up-regulated in the human brain in response to ischemic insult. Panel I. Representative staining of thin sections of cortical brain tissue derived at autopsy from patients whose cause of death was diagnosed as either ‘sepsis and prematurity” (top row) or “extensive germinal matrix hemorrhage with interventricular extension and infarction” (bottom row). General cellularity and morphology is revealed through H and E staining (a,d). Adjacent sections stained for ZEB1 protein expression shows a clear up-regulation in the developing cortical region in the patient diagnosed with hemorrhagic ischemia (e), in a pattern similar to that seen in the experimentally-induced ischemic cortex of the P7 rat pup (Figure 1, panel II). Magnification of the corresponding sections demonstrates the nuclear localization and intensity of ZEB1 staining in the ischemic tissue (f), while levels in the non-ischemic brain remain at background (c). Scale bars = a,b,d,e. = 500 µm, c,f. = 50 µm. Panel II. Chi-squared analysis of the data indicates that the association of ZEB protein induction with the diagnosis of an acute ischemic episode is highly significant.

Lastly, we investigated whether the ischemia-mediated increase in ZEB1 protein levels has a clinical correlate by staining (in a blinded fashion) archived cortical tissue obtained from autopsies of patients having a clear diagnosis of an acute ischemic episode, compared to those presenting with no such evidence. Representative staining patterns derived from two individuals are shown in **Figure 8A**. The intensity and localization of ZEB1 staining in the cortex from the premature infant diagnosed with gross cranial hemorrhagic ischemia (**bottom row**) is very similar to that seen in the P7 rat cortex (**Figure 1, panel II**). In contrast, the staining intensity in sections derived from the cortex of a patient having no evidence of ischemia-based neuropathy was relatively faint (**Figure 8A, top row**). The statistical results derived from a total of 71 different patient samples, demonstrated a highly significant association between ZEB1 protein induction and a diagnosis of an acute ischemic episode (**Figure 8B**).

## Discussion

A clearer understanding of the pro-survival signaling pathways and their downstream targets triggered by ischemia in cells of the cortical parenchyma is critical to the eventual development of therapeutic interventions to mitigate permanent neurological damage. The data presented here provide the first evidence that the transcriptional repressor ZEB1: 1) is dramatically induced in response to experimentally-administered permanent stroke, as well as in the human cortex in response to clinically relevant acute ischemic episodes; 2) can protect primary neuronal cultures against OGD and other pro-death insults; 3) decreases neuronal vulnerability to OGD in a dose-dependent fashion; 4) is itself transcriptionally up-regulated by p63 in this pathological context; 5) may inhibit, mitigate or delay neuronal apoptosis in an acute time frame by repressing TAp73. The stroke-induced increase in ZEB1 mRNA and protein levels in cortical neurons is both developmentally and phylogenetically conserved and may therefore be part of a fundamental cellular response to this insult. Beyond the context of stroke, the finding that ZEB1 is regulated by a member of the p53 family has implications for cell survival in other tissue and cellular environments subjected to ischemia, such as the myocardium [for review, see 32] and, in particular, tumor masses [for review, see 33]. This is the first report ascribing a role for ZEB1 in cellular survival, substantiating a recent report by Grooteclaes et. al., showing that one of ZEB1's co-repressors, CtBP1, itself has anti-apoptotic activity [24, and see below]. In addition, to our knowledge, this is also the first report to suggest a role for p63 in cell survival in the ischemic CNS. Finally, these data provide a plausible mechanistic basis for the phenotype of the proportionately undersized ZEB1 knockout mouse.

While our understanding at the molecular level is incomplete, the well documented cellular responses to ischemia include the rapid depletion of ATP stores with a concomitant shift in the redox state, creating an overall reducing environment that affects virtually all aspects of cellular metabolism and physiology, from gene expression patterns to energy use and production [5], [6], [34]. Using cell culture conditions similar to those we employed here, Zhang et. al. recently demonstrated that a ZEB1 co-repressor, CtBP1, can, by virtue of its use of NAD(P)H as a co-factor, sense very subtle alterations in the cellular redox potential, as manifest in the ratio of NAD(P)+ to NAD(P)H. Binding of NAD(P)H to CtBP1 significantly increases the affinity of CtBP1 for ZEB1, increasing proportionately the ability of this complex to repress target genes [22]. In light of these data, our finding that ZEB1 protein levels increase by an order of magnitude in rat cortical neurons in response to the reducing environment created by permanent FCI (**Figure 1, panel II**), as well as the human cortex in response to acute ischemic episodes (**Figure 8**), suggests that multiple pathways converge to prevent ZEB1-target genes from being activated. We note here that protein levels of the other, well-characterized member of the zinc-finger homeodomain (ZFHX1) family, Smad-Interacting Protein 1 [SIP1, ref. 44] was, itself, up-regulated, in both juvenile and adult rats, an average 2-3-fold in the ischemic cortex in our permanent stroke model (data not shown). Over the last several years, a number of groups have described the involvement of ZEB1 (ZFHX1A) and SIP1 (ZFHX1B) – both individually and cooperatively – in the transcriptional repression of the E-cadherin gene during the epithelial-to-mesenchymal transition of carcinoma cells subjected to the low oxygen/ischemic/reducing environment in the interior of a growing tumor mass (a necessary step in the progression of tumors towards metastasis, [refs. 51]–[54]). We are currently examining the relative contribution that each of these two closely related transcriptional repressors makes, individually and together, to cellular survival in response to hypoxic/ischemic challenge.

In light of the entirety of the data presented here, it will be clearly important to know not only how long ZEB1 induction persists beyond the 72 hr period we observed histologically (in both the neonatal and adult rat cortex, data not shown), but to map out the regional and cellular specificity of this induction with respect to the expanding ischemic lesion. Further, in the context of a mild ischemic episode, or experimental pre-conditioning, is ZEB1 induced, and does evidence of persistent induction correlate with the protective effects of these mild insults?

The importance of maintaining certain ZEB1-target genes in the off state in this context is underscored as well by our finding that primary cortical neurons isolated from ZEB KO mice are over twice as sensitive to OGD as their wt counterparts (**Figure 2**). This is further reinforced by a recent microarray-based study from the laboratory of Frisch (in the context of tumor progression), demonstrating that CtBP1 can target a number of epithelial-specific and pro-apoptotic-specific genes, and, potentially, play a key role in the epithelial-to-mesenchymal transition in the relatively hypoxic interior of a tumor mass [24].

Over-expressed ZEB1 was able to protect cortical neurons in culture from a number of biologically-relevant insults with the exception of the widely studied neurotransmitter/regulatory molecule nitric oxide (**Figure 3**). This result is important not only because of the well established role of NO in a wide variety of normal and patho-physiological processes, including many neurodegenerative diseases and stroke [47], [48], but as well may offer a clue about the mechanistic basis for ZEB1's neuroprotective activity. At lower or physiological levels, NO itself has been shown to be neuroprotective, while intermediate and higher levels can cause apoptosis and necrosis, respectively [45], [46], [49]. In addition, as with any bio-active agent, the ultimate effect of NO will depend on many factors, such as neuronal cell-type, age of the culture, percentage of glial cells, media components, source and half-life of the NO-donor, cellular redox state, etc.

Regardless of the experimental conditions, it is interesting that ZEB1 could protect our cortical cultures from an insult – excitotoxicity – which is known to trigger an nNOS-mediated increase in intracellular NO/reactive nitrogen species, but not from a direct challenge with nitric oxide itself (**Figure 3** and see corroborating data in **Figures S3, S4** and **S5**). One possible explanation for this result is that the levels of reactive nitrogen species generated in our excitotoxicity experiments fell below the threshold that can overcome ZEB1 protection – a level clearly attained in the experiments in which an NO donor was administered alone. Primary cultures of cortical neurons do not become sensitized to excitotoxic insult until they have expressed at least a threshold level of NMDA receptors, about 14 DIV [50]. To minimize the possible influence of culture-derived excitotoxic effects (e.g. NMDA receptor mediated induction of nNOS, [refs. 47], [50]) primary cortical neurons were challenged with the NO donor between 4 and 6 DIV. As ZEB1 can protect against reactive oxygen species (ROS) derived from administration of H_2_O_2_ to these cultures, it is possible that under the conditions we employed here, the ROS generated as a result of excitotoxic challenge predominates over NO-derived reactive nitrogen species.

While the NO-donor compound, S-nitroso-glutathione (SNOG/GSNO), had clear neuro-toxic effects at the concentration employed here (250 µM), it's relatively long half-life (compared to other NO donors) may have contributed both to it's toxicity, and it's potential for non-physiological nitroslyation. Nitric oxide-mediated nitrosylation of cysteine sulfhydryl groups has been shown to occur on and regulate the activity not only of NMDA receptors, but of various other ion channels, signaling intermediates, enzymes and transcription factors [48]. It is therefore possible that NO could nitrosylate ZEB1 itself (for example, on cysteine residues in the zinc-finger DNA-binding domains) disrupting it's functional (and hence, neuro-protective) activity by, for example, disrupting its binding capacity/specificity, etc.). Even if such a modification were a normal regulatory component of ZEB1 activity, it would likely be amplified to a patho-physiological extreme as a consequence of mass action resulting from the experimental introduction of high levels of both NO (via addition of an NO donor) and ZEB1 (via transfection of an expression vector). Similar non-physiological nitrosylation reactions have been shown in some cases to cause recipient protein mis-folding and precipitate an apoptotic cellular response [46], [48]. Such a covalent modification could, as well, simply result in a loss of function (e.g. a regulatory off switch) that reduced or eliminated ZEB1's neuroprotective capacity. Further experimental approaches are clearly warranted to determine the mechanistic basis for the ZEB1 – NO relationship.

The p63→ZEB1⊣p73 transcriptional cascade described here parallels recent results demonstrating a role for another well-characterized E-box-binding zinc-finger transcriptional repressor, SLUG, in protecting hematopoietic precursor cells subjected to low levels of ionizing radiation. In that case, radiation-induced p53 transcriptionally activates SLUG, which, after binding to the PUMA promoter, blocks subsequent p53-mediated up-regulation of that gene, allowing these cells to survive a relatively mild insult [35].

Our finding that the p63β mRNA is induced in response to ischemia to an extent far greater than the other isoforms (**Figure 4D**) is particularly interesting in light of the results of Jacobs et. al. in their recent analysis of growth factor-withdrawal-mediated apoptosis of sympathetic neurons [17]. In that report, withdrawal of nerve growth factor (NGF) caused the up-regulation of the gamma isoform of p63 (albeit with slower kinetics, 22 hrs after withdrawal before the visualization of p63γ protein induction), while beta isoform levels remained relatively constant. Whether stroke-mediated recruitment of the beta splicing variant is related to cell type (cortical neuron vs. sympathetic), or insult (ischemia vs. growth factor withdrawal), is unknown at present. Our analysis of the functional activity of p63 on the ZEB1 promoter showed that both the TAβ and ΔNβ isoforms could bind to and transactivate through their cognate site in the ZEB1 promoter. While TAp63γ did show binding activity, neither the TAγ nor ΔNγ isoforms showed any significant trans-activity (**Figure 5B and 5C**). As the antibody employed in the anti-p63 ChIP assay (monoclonal 4A4) recognizes an epitope common to all p63 isoforms, we do not know, as yet, which (or which combination) binds to the ZEB1 promoter in vivo.

Our results using SiRNA on primary cortical neurons derived from either wt or ZEB1-null mice (Figure 6) demonstrate that approximately 60% of OGD-mediated induction of ZEB1 can be attributed to p63. Surprisingly, the OGD-mediated induction of ΔNp63 (about seven-fold) is itself reduced by half in the absence of ZEB1 (Figures 6a and 6b), suggesting the existence of a positive regulatory feed-back loop between these proteins, though the mechanistic basis for this (ZEB1 represses a repressor of p63?) remains to be determined. In the aggregate, these data not only reinforce the existence of the ischemia/OGD-mediated regulatory links we have established here between p63, ZEB1 and p73, but imply a further feedback loop between either ZEB1 or p73 and p63.

In the hours immediately following the onset of ischemia, our data indicate as well that the protein levels of the (generally) pro-survival ΔN isoforms of both p63 and p73 increase relative to the (generally) pro-apoptotic TA isoforms (**Figures 4C, 4D** **and** **6A**). While the mechanistic basis for this is at present unknown, our data suggest that the ischemia-mediated transcriptional repression of TAp73 by ZEB1 may be critical to establishing a ΔNp73/TAp73 ratio that favors, at least in an acute time frame, a pro-survival outcome.

Although the mechanism(s) of cell death – apoptosis, necrosis, autophagy, or a hybrid or combination of these – remains a matter of debate, an emerging consensus in the literature maintains that apoptosis plays a more significant role in ischemic injury in the developing brain, with necrosis becoming the more predominant – though not exclusive – form in the mature brain [6], [7], [8]. At either stage of development, we have demonstrated here that the ischemia-mediated induction of a novel neuro-protective protein – ZEB1 – is an early event, occurring as early as 90 minutes after the ischemic insult (**Figures 1** **and** **4**).

As in the case of the well-studied p53-regulated response to environmental insults, the ultimate fate of a neuron challenged with ischemia depends on whether the integration of signaling events and the subsequent genetic response exceeds the threshold required to precipitate a death response. Our finding that p63 is induced early in the ischemic cortex and initiates a transcriptional cascade that manifests, ultimately, in an increase in the ratio of pro-survival ΔNp73 to the pro-apoptotic TAp73, may provide one mechanistic basis for how a neuron might survive a mild ischemic episode. In a severe, prolonged stroke, this rapidly-induced cellular defense response (analogous to the parallel up-regulation in neurons of the pro-survival Erythropoietin receptor [36]), will, of course, be overwhelmed by the weight of pro-death signaling (as well as the depletion of cellular ATP). In this case, our data [and that of others], [15], [16, 17] imply that one major influence on neuronal fate in the ischemic CNS will be the effective ratios between the pro-survival and pro-apoptotic isoforms of both p63 and p73, and suggests that ZEB1 plays a role in the transmission of this survival response. Based on our current detailed understanding of how p53 family isoforms can transcriptionally cross-regulate and hetero-tetramerize with one another [for review, see 37], however, it is likely that the regulatory cascade we describe here is far more complex. A direct role for the ΔN isoform of p63 in the repression/inhibition of pro-apoptotic p73 has recently been described in the context of squamous cell carcinoma [30].

The findings presented here raise several questions. First, is ZEB1 involved in the well-documented protective effect conferred on the CNS, myocardium, and other tissues by ischemic pre-conditioning [38], [39]? Second, in addition to permanent FCI, does ZEB1 play a role in other types of hypoxic-ischemic-mediated injury, in particular ischemia/reperfusion injury (i.e. in the context of increased reactive O_2_ species in vivo) and traumatic brain injury, as well as in other neuro-degenerative diseases? In this regard, similar to the regulatory pathway described above involving the transcriptional repressor SLUG, [35], it is possible that, in a certain cellular and patho-physiological contexts, ZEB1 could be a direct target of p53 itself. Third, in addition to p63-mediated transcriptional induction, what other mechanisms, transcriptional or otherwise, play a role in vivo in the dramatic ischemia-mediated increase in ZEB1 protein levels (not to mention the mechanistic basis for the rapid induction of p63). Lastly, in addition to p73, which other pro-death genes/pathways are targeted by ZEB1?

We have presented compelling evidence that ZEB1 induction is part of a protective response by neurons to ischemia. The fundamental nature of this pathway in cortical neurons is underscored by its phylogenetic and developmental conservation. The extent to which this survival pathway is utilized by other tissues and/or in other patho-physiological contexts remains to be seen.

## Methods

### Induction of permanent focal cerebral ischemia

All procedures and manipulations involving animals were carried out in accordance with Emory University Institutional Animal Care and Use Committee guidelines. The procedure, employing either a suture embolism (sized in proportion to the weight of the animal) of the mid-cerebral artery (MCA) in P7 rat pups, or electro-coagulation of that artery in adult rats, has been described in detail [23], [40]. Animals were sacrificed and perfused as described [40], and brains harvested for processing at 1, 1.5, 3, 6, 12, 24, 48, and 72 hrs after induction of FCI.

### Isolation of primary cortical cultures

Primary cultures of rat or mouse cortical neurons were isolated as described [41] and plated onto poly-D-lysine-coated (60 µg/ml) coverslips or tissue culture wells. Cortical neurons derived from ZEB1 KO mice were plated onto dishes coated with both poly-D-lysine and laminin (10 µg/ml, SIGMA # L2020). All primary cortical cultures were used from 4 to 6 DIV (days in vitro), with the exception of neurons used for excitotoxicity studies, which were cultured for 14–18 DIV before excitotoxic challenge.

### Isolation of total RNA

Dissected cortices were briefly rinsed in sterile PBS, immediately homogenized in Tri-reagent (SIGMA), and total RNA isolated according to the manufacturer's protocol. Total RNA was isolated from tissue culture cells using the RNAqueous-4PCR kit (Ambion) according to their protocol.

### RT-PCR

50 ng of DNAse-treated total RNA isolated from normal, sham, ischemic, or control P7 rat cortexes was used in 50 µl reaction with of the primer pairs (0.6 µM final concentration; for primer sequences see supplemental procedures) in a one-step RT-PCR reaction buffer (Qiagen) according to the manufacturers protocol. Cycling parameters for all samples were as follows: 50°, 30 min., one cycle; 95°, 15′, one cycle; 94°, 1 min.; either 58°(TAp63, p63β and ΔNp73) or 60°(ZEB1 and ΔNp63) or 65° (p63α, p63γ,TAp73 and β-actin); 72° 1 min., 35 cycles; 72°. 10 mins, one cycle. To control for the presence of contaminating genomic DNA, a PCR-only reaction using identical conditions was carried out (-RT lane, **Figure 2b**) using these samples. For positive controls, 0.1 ng of plasmid DNA harboring the full-length cDNA for a particular p63 isoform was used as the template in an identical reaction.

### Isolation of protein

Tissue samples from cerebral cortex were removed and homogenized in a lysis buffer containing 20 mM Tris [pH 7.4], 150 mM NaCl, 1 mM EDTA, (TNE Buffer) 1 mM EGTA, 1% Triton, 0.1% SDS, and a protease inhibitor cocktail (PI), comprising 5 mM phenylmethylsulfonyl fluoride, and 10 µg/mL each of the protease inhibitors antipain, chymostatin, pepstatin, and leupeptin. Samples were then sonicated for 3×15 s and cleared by centrifugation at 12,000×g for 20 min at 4C°. For isolation of total protein from cultured cells, homogenates were prepared in TNE buffer (with PI), 1% SDS, 0.25% Na_2_Deoxycholate, and 0.25% NP-40, and cleared as above. The protein concentration of the supernatant was measured using Protein Assay Reagent (Bio-Rad).

### Western blotting

Western blots were performed using standard protocols [41], [42]. To visualize ZEB1 protein, which, due to its net negative charge (pI = 4.73), has a significantly reduced electrophoretic migration rate, samples were loaded onto 4–20% gradient gels. After transfer of protein to nitrocellulose, blocking, incubation with appropriate antibodies and extensive washing steps, target proteins were visualized using chemilumenescence (Pierce). Membranes were stripped using Strip-EZ reagent (Pierce), tested for absence of signal, and re-probed and developed as above.

### Recombinant DNA constructs, PCR primers, and Oligonucleotides

All recombinant DNA expression and reporter constructs were synthesized and purified according to standard protocols [42]. Oligonucleotides (with or without modifications) were obtained from Integrated DNA Technologies (IDT, Coralville, Iowa). Correct reading frames and sequences for all recombinant constructs were verified by sequencing in both directions at Emory University's DNA Sequencing Core Facility.

The murine ZEB1 cDNA was fused in frame with GFP at its carboxyl end, to create pCMVZEB-GFP, by synthesizing a full-length ZEB1 PCR product with Xho I and Bam HI restriction sites at the 5′ and 3′ ends, respectively; Top 5′– CGACTCGAGCCTCGAGCCACGACCATGGCGGATGGCCCCAGGTGTAAGCG-3′; Bottom 5′-CCACGTCTCGGATCCAGCTTCATTTGTCTTCTCTTCAGACAGCTGC-3′, and inserting the gel-purified digestion product into the CMV promoter-driven expression vector pEGFP-N1 (CLONTECH). PCR synthesis of mouse ZEB1 promoter fragments were carried out using the bacterial artificial chromosome RP23-345J21 (BACPAC Resources, Children's Hospital Oakland Research Institute, Oakland, CA) as a template, and the following primer sets: pmZPRO1300LUC, Top, 5′-CAGTCAAGCTTGGGAGAAGGGTCAACTTAAGTCCCTA-3′; pmZPRO700LUC, Top, 5′-CAGTCAAGCTTTGGCAAGTCACTACAACCCTGAGT-3′ the Bottom primer for both was 5′- CAGTCAGTCGACTCCTCTCGCTTGTGTCTAAATGCTCG-3′. The top primers incorporated a HindIII site, and the bottom a SalI site. The gel-purified PCR products were restriction digested, re-purified, and then cloned into the luciferase reporter plasmid pGL3-Basic (Promega). The p53-family binding site was mutated using the Quick Change II site-directed mutagenesis kit (Stratagene) and a double-stranded primer pair with the sequence: TOP-5′ catctggaataacaa AcGgcAtCgA cActTgaCgT caacaataaaaggac (plus the reverse complement), (p53 binding site is underlined with mutated bases in lower case). Primer pairs crossing intron 1 of the mouse ZEB1 gene for RT-PCR analysis were as follows: Top, 5′-TGTAAGCGCAGAAAGCAGGCGAA-3′; Bottom, 5′-GTCACTGCCTCCTGGTAATACTGT-3′; for 18S rodent rRNA: Top, 5′- CGGCTACCACATCCAAGGGAA -3′, Bottom, 5′- GCTGGAATTACCGCGGCT – 3′.

For the ZEB promoter ChIP analysis, primers encompassing the p53 binding site in the rat ZEB1 promoter region (−1160 to −778 relative to the transcriptional start site, yielding a 382 bp product) were: Top, 5′- GGAAAGATCTCCCATGCTCATGG -3′, Bottom, 5′ CCATTGGATGGTTTTGGCTCCTTTG -3′. For the human p73 promoter ChIP analysis, primers flanking five ZEB1 binding sites [31] in the human p73 intron 1 promoter region (−965 to −377 relative to the transcriptional start site, yielding a 588 bp product) were: Top, 5′- GGGACCTGAGCCACCTCCAGGTCCCGG-3′, Bottom, 5′- CGGTGGACTGGGCCATCTTCCCCACGCC-3′.

Primers used in the RT-PCR analysis of p63 isoform expression in the ischemic cortex: TAp63, Top, 5′- GCTGTTTCATAGAAACCCCATCTCATTTCTCCTGG-3′, Bottom, 5′-GCT GTGTGGGCCTGGGTAATCTGTGTTGGAGGG-3′; DeltaNp63, Top, 5′-CCTGGAAAGCAA TGCCCAGACTCAATTTAGTGAGCC-3′, Bottom, Same as for TAp63. Top primer for p63alpha, p63beta, and p63 gamma, 5′- AAGAGACCGGAAGGCCGATGAAG-3′; p63alpha, bottom, 5′- ACGGGGTGGGAAAGAGATGGTC-3′; p63beta, bottom, 5- TCAGACTTGCCAA ATCCTGACA-3′; p63gamma, bottom, 5′- CTCTCCGGGACTCCACAAG. Beta-actin primers, Top, 5′- TCTGTGTGGATTGGTGGCTCTA-3′, Bottom, 5- CTGCTTGCTGATCCACATCTG-3′.

Double-stranded oligonucleotides used in the microtitre-plate-based p63-ZEB1 promoter binding assays were as follows: wt oligo, (biotinylated and incorporating the p53-family binding site from the ZEB1 proximal promoter), TOP, 5′-acaatAGGATAGCTAAAACTATCCTatctc-Biotin–3′ (p53 binding site is capitalized); wt competitor oligo, same as wt without the 3′ biotin; mutant oligo, TOP, 5′- acaat AcGgcAtCgA cActTgaCgT atctc-3′; non-specific oligo, TOP 5′- acaatATCGGCATATGCATCGAGCatctc-3′.

### Histology

#### Antibodies used in these studies: I. Primaries

A rabbit polyclonal antibody specific for ZEB1 was generated and affinity purified commercially (New England Peptides, Inc.) using the following peptide: Ac-LETNQASLASKEQEAVS-Amide (corresponding to amino acids 409 to 425 of the mouse ZEB1 protein). The mono-specificity of this antibody was verified by the presence of a single band on western blots of whole brain lysates from both mouse and rat, (which could be competed away using in vitro-translated full-length ZEB1 protein [not shown]), as well as by the experiment described in Figure **S1**. For immunostaining paraffin sections the anti-ZEB1 Ab was used at 1∶000 dilution, for frozen sections, 1∶600, for western blots, 1∶5000, for chromatin immunoprecipitation, 1∶400. NeuN: Chemicon, MAB377, at 1∶250 dilution; active Caspase 3: Cell Signaling Technology, at 1∶500; p63, for immunostaining and chromatin immunoprecipitation assays: the pan-anti-p63 monoclonal Ab 4A4, Novocastra, 1∶250, for western blots, p63TA isoform: Santa Cruz, sc-8608, at 1∶500, p63DeltaN isoform: Santa Cruz, sc-8609, at 1∶750; p73, for immunostaining: Santa Cruz, sc-7957, p73TA isoform, Calbiochem OP108, at 1∶450, p73DeltaN isoform, Abcam ab13649, at 1∶750; PUMA, Cell Signaling Technology, at 1∶800; β-actin, Santa Cruz sc-1615 at 1∶1000; α-Tubulin, Santa Cruz sc-23948. II. **Secondaries**. For colorimetric immunostaining, a biotinylated horse radish peroxidase-coupled secondary antibody appropriate for the particular primary was used at 1∶100 dilution: Vector Labs, ABC Elite Staining Kit. For double-immunofluorescence staining, an Alexa-Fluor-coupled secondary antibody appropriate for a particular primary Ab was employed, green: Alexa 488, red: Alexa 594, all from Molecular Probes/Invitrogen. For western blotting, stabilized secondary antibodies were purchased from Pierce: anti-rabbit,1∶500,000 dilution; anti-mouse, 1∶200,000).

### Immunostaining

10 µm frozen coronal sections derived from formalin-fixed P7 or adult rat brains were prepared as described [40]. The procedure for staining paraffin sections of human autopsy brain tissue was identical to that described above for frozen sections, except they were subjected to de-waxing, re-hydration, and antigen unmasking (3×5′ at full power in a 750 Watt microwave in 10 mM Na_2_ citrate) before the permeablization step. Following a 20′ permeablization step (0.2% Triton-X 100 in PBS) and blocking of endogenous peroxidase (4% H_2_O_2_, 50% methanol) non-specific binding sites were blocked (3% BSA, 5% goat serum in PBS), and incubated with primary antibody in 0.5× blocking buffer supplemented with 0.1% Triton–X 100 overnight at 4°. After three 15′ washes in PBS, sections were incubated with secondary Ab in the same buffer used for the primary for 1 hour at RT. Following three 15′ washes in PBS, antigen was visualized using the chromogenic substrate 3-3′ diaminobenzi-dine (for HRP-coupled secondaries). For quantification of colorimetric staining of tissue sections or fixed cells, densitometry was performed on scanned, digitized images using the Image J software program (NIH). The use of archived pathology samples of human tissue for immunostaining in these studies was exempted by the Emory University School of Medicine Institutional Review Board (# 679-2004). The procedure for staining paraffin sections of human autopsy brain tissue was identical to that described above for frozen sections, except they were subjected to de-waxing, re-hydration, and antigen unmasking (3×5′ at full power in a 750 Watt microwave in 10 mM Na_2_ citrate) before the permeablization step.

### Treatment of Primary Cortical Cultures with Pro-death Insults

To test the ability of ZEB1 to protect primary cortical neurons (4–6 DIV; in the case of excitotoxicty challenge, 14–16 DIV) from death-inducing insults, cells were transfected with either EGFP alone or the ZEB1-EGFP fusion protein (Lipofectamine 2000 (GIBCO) at a ratio of 0.8 mg DNA∶4 ml reagent per well of a 12-well plate, according to the manufacturers protocol), and returned to the incubator for 24 hrs. At this time, the following death/apoptosis-inducing agents were used at the concentrations or doses specified: hydrogen peroxide, 75 µM final concentration; Glutamic acid, 500 µM final, made fresh; the nitric oxide (NO) donor, S-nitrosoglutathione (GSNO/SNOG), 250 µM final; TNF-α, 10 ng/ml final; a 10 Grey dose of gamma ionizing radiation (IR) generated from a Cesium source; or DMSO vehicle. Neurons kept between 14 and 16 DIV were subjected to oxygen-glucose deprivation by changing the normal neurobasal-based plating media to osmotically-balanced salts without glucose (116 mM NaCl, 5.4 mM KCl, 0.8 mM MgSO4, 1.8 mM CaCl2, 20 mM HEPES-NaOH, pH 7.4, sterile-filtered) and then placing them in a humidified, gas-impermeable chamber (Billups-Rothenberg, Inc.), and introducing a 95%N_2_/5%CO_2_ gas mixture into the inflow port until an oxygen sensor (PROOX 110, BioSpherix, Ltd.) attached to the outflow port registered less than 0.5% oxygen. The sealed chamber was then placed at 37° for the duration of the experiment. To prevent even momentary re-oxygenation (re-perfusion) of the cells upon their removal for harvesting or further processing, the chamber was placed in a gas-impermeable glove box and opened under anoxic conditions.

Following the various treatments, neurons were returned to 37° (in the case of the H_2_O_2_ treated cells, media was switched after 30′ to normal; the OGD-treated cells were returned to an O_2_-depleted atmosphere as described below), and individual coverslips were removed at 0′, 30′, 1 hr, 2 hr, 4 hr, and 8 hr, and 16 hr., to assess the relative cellular damage. To measure mitochondrial membrane integrity (**Figure 3, bottom**; and **S4**), cells were processed using the Image-iT LIVE kit (Molecular Probes/Invitrogen, according to the manufacturer' instructions. This method employs the cell-permeable Mitotracker Red CMXRos dye, that is only taken up and retained by mitochondria with an intact membrane potential. Though not dispositive (cells losing mitochondrial membrane potential either through the opening of the permeability transition pore or through other means have been shown to recover from apoptotic insults), neurons taking up and retaining this dye were scored as “healthy” compared with their dye-negative counterparts, by counting ten separate fields on each slide by two individuals in a blinded fashion. To assess the relative health of nuclei from these cells (**Figure 3, top**; and **S3**), parallel cultures were stained with Hoechst 33342 dye (Molecular Probes/Invitrogen) and neurons with pyknotic/shrunken/fragmented nuclei (a hallmark of dying cells) as compared with “healthy” nuclei (normal, rounded morphology, indistinguishable from untreated control cells) were scored in a similar fashion as with the mitochondrial dye. Cells were scored for the presence of double-stranded DNA breaks employing a rhodamine-coupled detection assay (TdT-based TUNEL-staining kit, Roche). TUNEL-positive cells were scored by dividing the GFP-rhodamine double-positive cells by the total number of rhodamine-positive cells in a given field. In all cases, ten separate fields were counted on each slide by two individuals in a blinded fashion (**Figure S5**).

### Transfections/Transductions/Luciferase Assays

Primary cultures of cortical neurons were transiently transfected with Lipofectamine 2000, and the osteosarcoma cell line SAOS-2 (**Figure 5b**) was transiently transfected with FUGENE 6, each according to the manufacturers instructions. Neurons were transduced with lentiviral constructs (amplified and concentrated at the Emory University Department of Neurology's Center for Neurodegenerative Disease's Viral Vector Core) at an M.O.I of between 1 and 5, depending on the construct. We routinely achieved transduction efficiencies of between 90% and 95%. SAOS-2 cells were lysed and assayed for reporter firefly Luciferase activity using the Luciferase Assay Reagent Kit (Promega), with a Renilla Luciferase reporter construct as the normalization control.

### Genotyping embryos

To genotype mouse embryos (or primary neurons in culture), a small portion of tissue from each embryo was aseptically removed at the time of dissection (taking care to eliminate cross-contaminating tissue from the dam or littermates), and genomic DNA isolated according to standard protocols [42]. To test for the presence of the wt ZEB1 allele, 1 ng of genomic DNA was subjected to the following PCR protocol: 94° for 3 minutes, for one cycle; 94°, 1 minute, 62°, one minute, 72°, one minute, for 37 cycles; 72°, 10 minutes, one cycle, using the following primers: Top, 5′ –ATGTCGTAAAGCCTCGAGTGTCGT – 3′ Bottom, 5′ - AGCAAGAGTGGAGAAAGTGCGGAA - 3′, the resulting 526 reaction product visualized on a 2% agarose gel. A separate PCR reaction was carried out to test for the presence of the LAC-Z cDNA (knocked into the ZEB locus [19],: 94° for 3 minutes, one cycle; 94°, 1 minute, 65°, one minute, 72°, one minute, 35 cycles; 72°, 10 minutes, one cycle, using the following primers: Top, 5′ – TAGGTGTTAGGAAGGTGATGTCG – 3′; Bottom, 5′ – AACCGTGCATCTGCCAGTTTGAG - 3′. The resulting 389 bp product was also visualized on a 2% agarose gel.

### Quantitative RT-PCR

The ZEB1 primer pair (TOP: 5′ - TGTAAGCGCAGAAAGCAGGCGAA - 3′; BOT: 5′ – ACAGTATTACCAGGAGGCTGAGAC – 3′) produced a single PCR product of expected size - 204 bp - using total RNA isolated from E16.5 or P7 rat cortex as a template, and no product using a 1 µg of a plasmid harboring a full-length cDNA encoding the closely-related SIP1 cDNA (a kind gift from D. Huylebroeck, [39]) as the template; the control SIP1 primer pair (TOP, 5′ - CAATGACTATAAAGTTCTTATGGCAACACATGGGTTTAGTGG – 3′; BOT: 5′ - CCGACAGGGGGAATATTGGGAGAAGTTACCCCTTGTTCT), produced a unique 498 bp product (see **Figure S6**); the 18S rRNA primer pair yielded an amplicon of 181 bp. Starting with either cortical- or primary cortical cell culture-derived total RNA (isolated using the RNAqueous Kit and DNAse I-treated with DNA-free reagent, both Ambion) as a template, RT-PCR was performed using a SYBR Green-based one-step protocol (QuantiTec SYBR Green RT-PCR kit, Qiagen), in a SMART-CYCLER II PCR machine (Cepheid, Inc.) using the following run parameters: RT step, 50°, 30 minutes; PCR step, 95°, 15 minutes, one cycle, 95°, 5 seconds, 60°, 15 seconds, 72°, 30 seconds, 45 cycles. The reaction mixture (25 µl) included 10 ng total RNA (determined by absorbance at 260 nm in H_2_O), 12.5 µl 2× Quantitect SYBR Green RT-PCR Master Mix, and 500 nM each primer. For either set of primer pairs, melting curve analysis (60° to 90° at 0.2°/sec) showed a single specific reaction product. For absolute quantification of ZEB1, RNA standards were synthesized by T7 polymerase-mediated in vitro transcription of the full-length mZEB1 cDNA (Megascript Kit, Ambion). Following treatment with DNase I to remove plasmid DNA, the integrity of the RNA was verified on an agarose gel, quantified spectrophotometrically (A_260_), and stored at a concentration of 10^10^ transcripts/ml at −80°. Serial dilutions of this standard, ranging from 50 to 5×(10)^6^ transcripts were run concurrently with each ZEB1 RT-PCR reaction above and used to generate a standard curve. This was used to convert the threshold cycle values from each run to ZEB1 message copy number based the weight of input RNA (normalized to 18S rRNA levels in a separate RT/PCR run under identical conditions, adjusting for a proportionate dilution of the input RNA to broaden the dynamic range of this analysis for this very highly abundant RNA). Samples were done in triplicate within each run and at least four separate runs were conducted per treatment. Excluding the outlier value of each triplicate, individual means from each run were determined and the combined mean number of transcripts+/−the S.E.M./µg input RNA was plotted.

### Microtitre plate-based analysis of DNA-protein interactions

The basic protocol derived from the No-Shift kit (Novagen, Inc.) was modified as follows. Full-length mouse p63 cDNAs for encoding TAp63α, ΔNp63γ (kind gifts of Frank McKeon); ΔNp63α and ΔNp63γ (kind gifts of Anil Rustgi); and TAp63β and ΔNp63β (kind gifts of David Sidransky and Barry Trink) were in vitro transcribed/translated using the TnT kit (Promega). Equimolar amounts of these full-length proteins for use in the DNA-binding assay were obtained by comparing, via densitometry, parallel TNT reactions (western blotted and visualized using a strepavidin-conjugated HRP chemi-luminescent detection system) incorporating biotinylated lysine residues (Transcend kit, Promega), to a standard curve of increasing amounts of a known biotinylated translation product (adjusting for the relative number of lysine residues in each full length translation product). Double-stranded oligos (with or without increasing amounts of competitor oligo) were first incubated for 1 hr on ice with approximately 50 ng of the TnT-generated protein (with appropriate binding buffer and competitor DNA from kit), bound to streptavidin-coated wells in a micotire plate for 1 hr at 37°, washed extensively, then incubated for 30 minutes at 37° with a 1∶500 dilution of anti-p63 (monoclonal 4A4, SantaCruz). Following incubation with a anti-mouse-HRP conjugated secondary Ab (diluted 1∶1000), a colorimetric substrate from the kit was added, allowed to develop for 25′, and read at A_450 nm_ in a plate reader (Synergy-HT, Bio-tek Corp.). (for sequences see **Recombinant DNA constructs PCR Primers, and Oligonucleoitdes**, above)

### Chromatin immunoprecipitation assay

E16–E17 derived mouse primary cortical cultures (isolated as described above and plated at 5×10^4^/ml) were subjected to OGD for the times indicated, approximately 1×10^6^ cells per time point per experiment (two, 10 cm plates). Normoxic controls and OGD-treated cells (without re-exposure to normoxia) were subjected to formaldehyde cross-linking (1% final, 20′) by direct addition to the tissue culture medium. Cells were harvested in ice-cold PBS, gently pelleted and re-suspended in 1 ml lysis buffer (5 mM PIPES, (8.0), 85 mM KCl, 0.5% NP40, plus protease inhibitors (PI) as above). After a second gentle spin (3000×g, 5′, 4°), cell pellet was resuspended again in 1 ml lysis buffer, and incubated on ice, 5′. Cells were re-spun (3000×g, 5′, 4°), supernatant discarded, and nuclear pellet resuspended in 0.5 ml nuclear lysis buffer (50 mM Tris-HCl (8.0), 10 mM EDTA, 1% SDS+PI), and incubated on ice for 30′, with vortexing every 10′. Debris was pelleted by spinning at 20,000×g for 20′, at 4° and the nuclear lysate was removed for shearing of DNA.

To process P7 rat pup brain, at a given time point following the administration of unilateral MCAO, animals were sacrificed, ischemic-side or sham-operated cortexes ware rapidly dissected out, placed in a weigh boat on ice, immediately minced into 1 mm pieces and transferred into a microfuge tube. Following cross-linking by incubating in 1% formaldehyde in PBS (15′, 37°), tissue was washed 5 times with ice-cold PBS. Tissue was then homogenized (Mini-homogenizer, Biospec Products, Bartlesville, OK) in 1 M sucrose, 10 mM Hepes [pH 7.9], 10 mM KCl, 3 mM MgCl2, (plus PI as above) and centrifuged in a microfuge at 2000 rpm, 10′, 4° to pellet cellular debris. The supernatant was then re-centrifuged at 11,000 rpm, 10′, 4°. The resulting nuclear pellet was reuspended in 10 mM Hepes [pH 7.9], 0.4 MNaCl, 3 mMMgCl2, 0.5 mM DTT, and incubated in ice for 30′, with vortexing every 10′. Debris was pelleted by spinning at 20,000×g for 20′, at 4° and the nuclear lysate was removed for shearing of DNA.

The ChIP procedure was carried out basically as described by Beresford, et. al. [43] with the following modifications. Nuclear lysates were sonicated using a Sonic-Dismembrator 60 (Fisher) fixed with a microprobe set at 50% power for 5×20 sec bursts on ice to an average chromatin fragment size of 600 bp (verified by running a reversed cross-linked aliquot on an agarose gel). 50 µl of the chromatin supernatant was pre-cleared by adding it to 950 µl of IP buffer (0.01% SDS, 1.1% Triton –X 100, 20 mM Tris-Cl (8.0), 1.2 mM EDTA, 167 mM NaCl) plus 100 µl of solution A (a 50% slurry of protein A Sepharose (Cl-4B, Amersham) in 10 mM Tris-Cl(8.0), 1 mM EDTA, 50 µl normal mouse serum, 0.2 mg/ml tRNA, 0.2 mg/ml salmon sperm DNA), and rotated 1 hr., 4°. After pelleting and discarding the protein A beads (3000×g, 5′, 4°), 5 µg of an appropriate antibody (either anti-p63 or anti-ZEB1) was added to the supernatant and the mixture rotated overnight at 4°. For negative controls, chromatin preparations were incubated with α-tubulin (heterologous, non-specific Ab) as well as without antibody. The following day, the immune complexes were immunoprecipitated by adding 60 µl of the above protein A slurry/BSA/SS DNA/tRNA mixture, rotating 1 hr, 4°, followed by gentle pelleting (3000×g, 5′, 4°). Supernatants were carefully removed, with the minus Ab control supernatant retained for use as a positive control in the subsequent PCR analysis. Pellets were washed by rotating for 10′ at 4°, with 3×1 ml of each the following buffers: IP buffer, TSE-500 buffer (0.1% SDS, 1% Triton X-100 2 mM EDTA, 20 mM Tris-HCl (8.0), 500 mM NaCl), LiCl buffer (100 mM Tris-Cl (8.0), 500 mM LiCl, 1% NP-40, 1% deoxycholic acid), and TE. Macromolecular complexes were eluted from the Protein A beads with 2×250 µl of EB (50 mM NaHCO_3_, 1% SDS). Formaldehyde crosslinks were reversed by incubating eluates (including 400 µl of the minus Ab negative control) at 65° o/n after addition of 20 µl 5 M NaCl. DNA was purified from the eluted complexes by the addition of Proteinase K, followed by phenol/chloroform extraction. After addition of carrier, chromatin fragments were EtOH precipitated, washed and resuspended in 30 µl water. One-tenth of this DNA solution (or 1/1000^th^ of the positive control supernatant) was used for PCR amplification (95°, 15 mins, one cycle, 95°, 5 seconds, 61°, 15 seconds, 72°, 30 seconds, 40 cycles). ChIP assays for p63 binding and ZEB1 binding were each carried out three times.

## Supporting Information

Figure S1 Specificity of the anti-ZEB1 antibody used in these studies. Full-length ZEB1 and the closely-related SIP1/ZEB2 35S-labeled in vitro translation products were separated via SDS-PAGE and immunoblotted using the anti-ZEB1 polyclonal antibody; left panel (labeled 35S), 24 hr exposure of the nylon membrane, revealing the 35S labeled protein bands; right panel (labeled WB), 5 min chemilumenescent exposure of the same blot after immunoblotting.
(0.24 MB TIF)

Figure S2 Double-immunofluorescence staining of rat P7 ischemic cortex 12 hours post-FCI. ZEB1-positive neurons and cells staining positive for either active Caspase 3 (Top row) or the BH3-only Bcl-2 pro-apoptotic family member PUMA (Bottom row) are mutually exclusive. Scale bar = 50 µm.
(1.45 MB TIF)

Figure S3 Over-expressed ZEB1 mitigates cellular damage/death in primary cortical neurons subjected to a battery of toxic insults: effect on nuclear morphology. Primary cultures of cortical neurons were co-transfected with either GFP alone (White Bars) or a full-length cDNA for ZEB1 fused to GFP (Blue Bars). Eighteen hours later cells were subjected to a battery of pro-death/toxic insults (for details, see Materials and Methods). GFP-positive neurons were processed and scored (in a blinded fashion) for having either a normal vs a pyknotic/mis-shapen/condensed morphology. In this example, representative photomicrographs of cells challenged with OGD for 6 hrs indicate that a greater percentage of ZEB1-transfected cells are over twice as likely to retain a rounded “normal” nuclear morphology, than those transfected with GFP alone (panels d and e). For every administered insult (except nitric oxide - see text) in an acute time-frame, and in a dose-dependant manner, nuclei of ZEB1-transfected neurons maintained a “normal”, rounded morphology. Results for six different insults are summarized graphically in Figure 3. In both panels, a. GFP alone; b. GFP plus Hoechst; c. Hoechst alone; d. and e. magnified Hoechst images from c. Scale bars, a–c = 50 µm; d,e = 10 µm.
(1.64 MB TIF)

Figure S4 Over-expressed ZEB1 mitigates cellular damage/death in primary cortical neurons subjected to a battery of toxic insults: effect on mitochondrial membrane integrity. Primary cultures of cortical neurons were co-transfected with either GFP alone (White Bars) or a full-length cDNA for ZEB1 fused to GFP (Blue Bars). Eighteen hours later cells were subjected to a battery of pro-death/toxic insults (for details, see Materials and Methods). GFP-positive neurons were processed and scored (in a blinded fashion) for either intact (rhodamine stain) or compromised (absence of rhodamine stain) mitochondrial membranes. In this example, representative photomicrographs of cells challenged with OGD for 6 hrs indicate that a greater percentage of ZEB1-transfected cells maintain mitochondrial integrity, than those transfected with GFP alone (panels h and i). For every administered insult (except nitric oxide- see text) in an acute time-frame, and in a dose-dependant manner, mitochondrial integrity in ZEB1-transfected neurons was maintained relative to neurons transfected with GFP alone. Results for six different insults are summarized graphically in Figure 3. In both panels, a, phase contrast; b. GFP-positive cells; c. TMRhodamine-red-positive cells, indicating intact mitochondria; d. Hoechst staining of nuclei; e. phase plus GFP; f. GFP plus TMR-red; g. GFP plus Hoechst; h. triple stain; i. magnification of indicated area in panel h. Scale bars, a–h = 50 µm; I = 20 µm.
(4.08 MB TIF)

Figure S5 Over-expressed ZEB1 protects primary cortical neurons from OGD-mediated cellular damage/death: time course of TUNEL-labeling. Primary cultures of cortical neurons were co-transfected with either GFP alone (White Bars) or a full-length cDNA for ZEB1 fused to GFP (Blue Bars). Eighteen hours later, cells were subjected to OGD for the times indicated, fixed under hypoxic conditions, and GFP-positive cells were scored for TUNEL staining in a blinded fashion. Consistent with the results from Figure 3, over-expressed ZEB1 reduced the average number of TUNEL-positive cells by less than half at the 12 hr time point. Results shown are the average of three separate experiments (cultures isolated on different days)+/−the S.E.M.; * = P<0.01 by Student's T-test.
(9.48 MB TIF)

Figure S6 Oxygen-glucose deprivation increases steady-state levels of ZEB1 mRNA in primary neuronal cultures. (A) Top Panel, Primers used in this analysis (see Materials and Methods for sequences) cross intron 1 of the mouse ZEB1 gene to yield a 204 bp PCR product. Bottom Panel, Control PCR reactions demonstrating ZEB1 primer specificity. Lane 1, 498 bp SIP1 PCR product derived using SIP1-specific primers and SIP1 cDNA template; lane 2, 1 µg SIP1 cDNA template with ZEB1-specific primers; lane 3, 204 bp product derived using ZEB1-specific primers with 1fg ZEB1 cDNA template; lane 4, 204 bp RT-PCR product using ZEB1-specific primers and 10 ng total RNA isolated from an E16.5 rat primary cortical culture; lane 5, same as 4 minus RT; M, 100 bp incremental markers. (B) Quantitative RT-PCR results using indicated ZEB1-specific primers and 10 ng total RNA isolated from E16.5-derived primary cortical cultures subjected either to normoxia or OGD (3 hrs. with RNA harvested under hypoxia). Values are numbers of transcripts of ZEB1 mRNA (derived from a standard curve generated concurrently in the same PCR run) per µg of input total RNA.
(0.45 MB TIF)
