# Supplementary figures and images for: ZEB1 Links p63 and p73 in a Novel Neuronal Survival Pathway Rapidly Induced in Response to Cortical Ischemia
Source: PLoS One. 2009 Feb 4;4(2):e4373. doi: 10.1371/journal.pone.0004373 (PMC2632749; doi:10.1371/journal.pone.0004373)

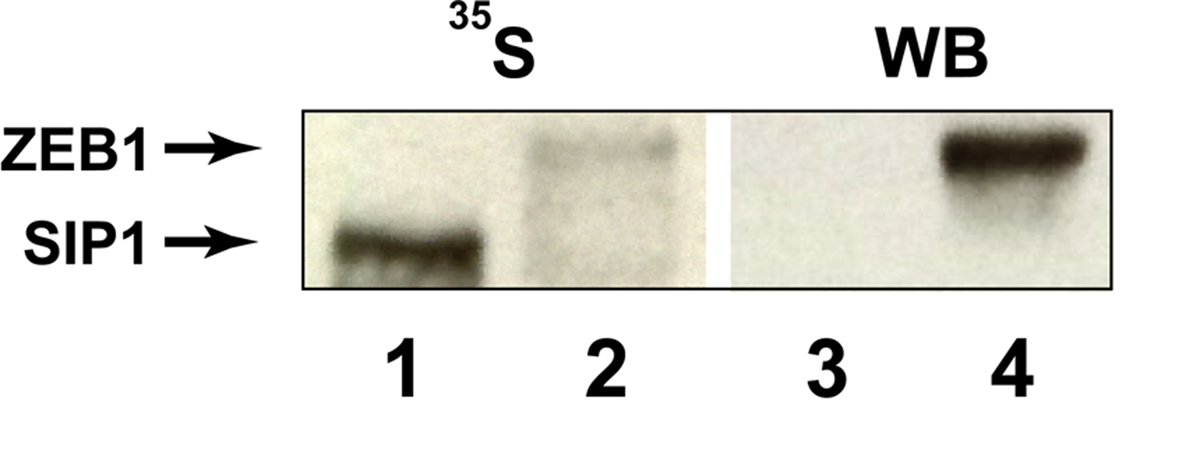

Supplement: Figure S1 — Specificity of the anti-ZEB1 antibody used in these studies. Full-length ZEB1 and the closely-related SIP1/ZEB2 35S-labeled in vitro translation products were separated via SDS-PAGE and immunoblotted using the anti-ZEB1 polyclonal antibody; left panel (labeled 35S), 24 hr exposure of the nylon membrane, revealing the 35S labeled protein bands; right panel (labeled WB), 5 min chemilumenescent exposure of the same blot after immunoblotting. (0.24 MB TIF) [file pone.0004373.s001.tif]

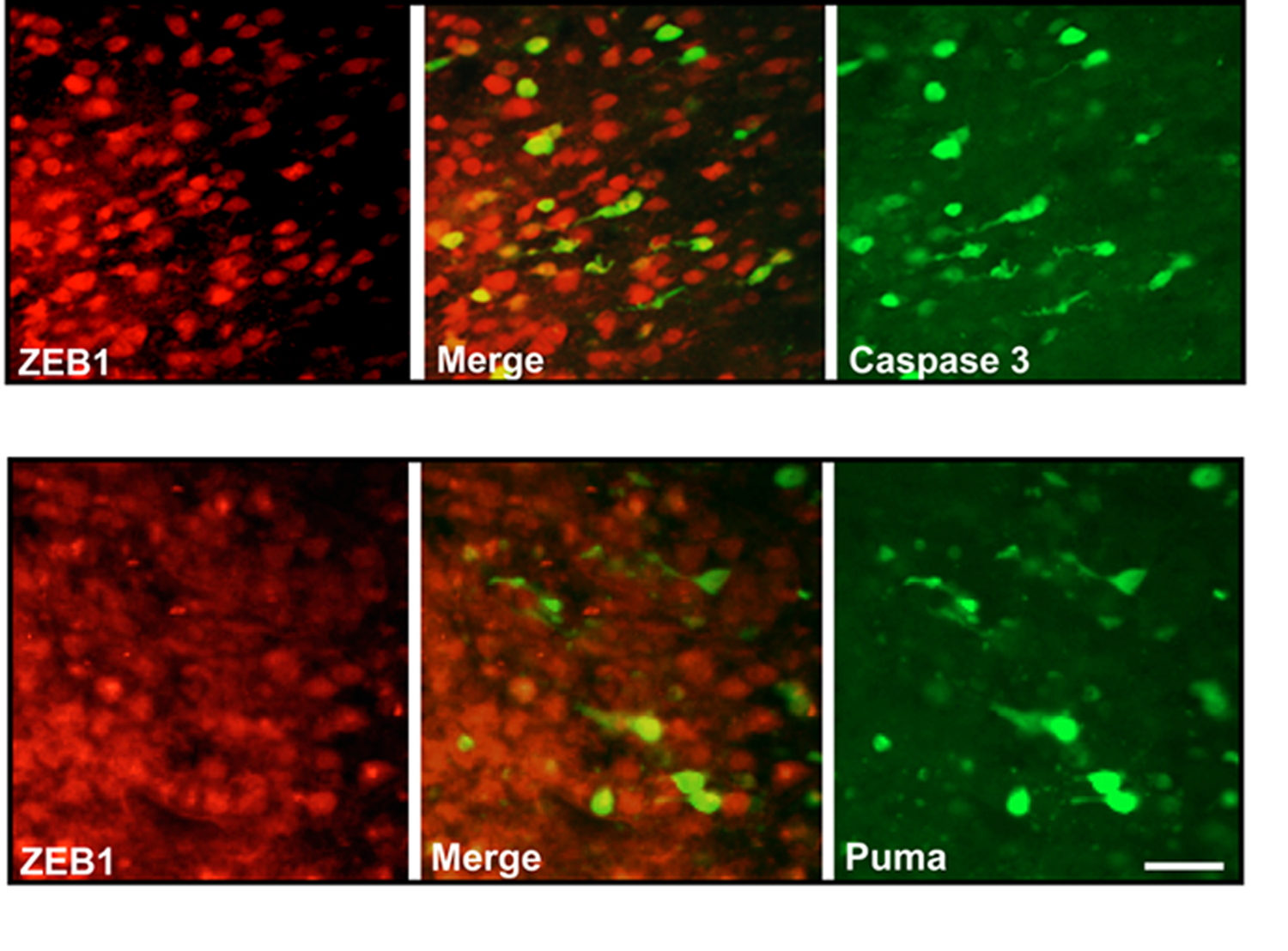

Supplement: Figure S2 — Double-immunofluorescence staining of rat P7 ischemic cortex 12 hours post-FCI. ZEB1-positive neurons and cells staining positive for either active Caspase 3 (Top row) or the BH3-only Bcl-2 pro-apoptotic family member PUMA (Bottom row) are mutually exclusive. Scale bar = 50 µm. (1.45 MB TIF) [file pone.0004373.s002.tif]

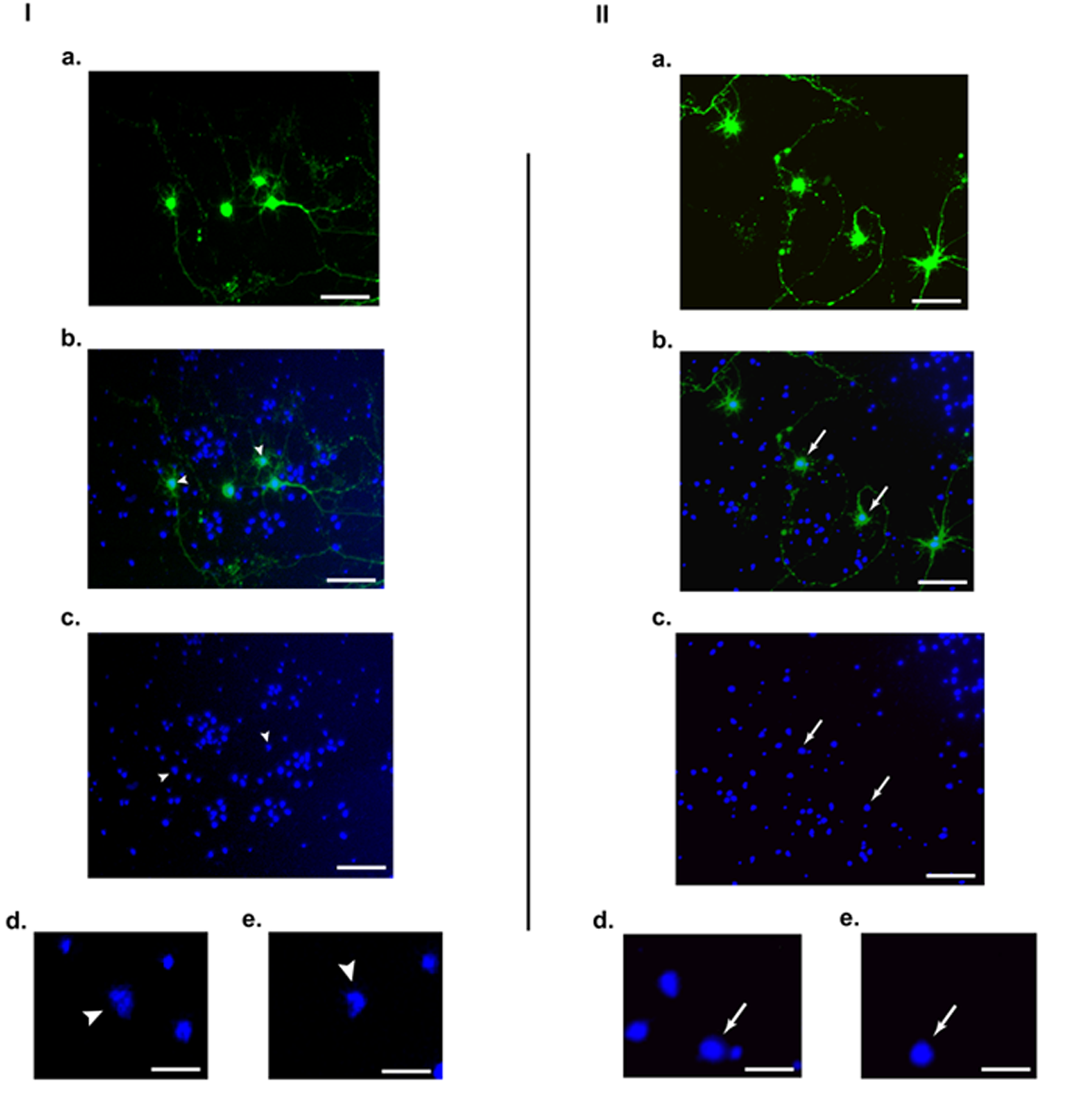

Supplement: Figure S3 — Over-expressed ZEB1 mitigates cellular damage/death in primary cortical neurons subjected to a battery of toxic insults: effect on nuclear morphology. Primary cultures of cortical neurons were co-transfected with either GFP alone (White Bars) or a full-length cDNA for ZEB1 fused to GFP (Blue Bars). Eighteen hours later cells were subjected to a battery of pro-death/toxic insults (for details, see Materials and Methods). GFP-positive neurons were processed and scored (in a blinded fashion) for having either a normal vs a pyknotic/mis-shapen/condensed morphology. In this example, representative photomicrographs of cells challenged with OGD for 6 hrs indicate that a greater percentage of ZEB1-transfected cells are over twice as likely to retain a rounded “normal” nuclear morphology, than those transfected with GFP alone (panels d and e). For every administered insult (except nitric oxide - see text) in an acute time-frame, and in a dose-dependant manner, nuclei of ZEB1-transfected neurons maintained a “normal”, rounded morphology. Results for six different insults are summarized graphically in Figure 3. In both panels, a. GFP alone; b. GFP plus Hoechst; c. Hoechst alone; d. and e. magnified Hoechst images from c. Scale bars, a–c = 50 µm; d,e = 10 µm. (1.64 MB TIF) [file pone.0004373.s003.tif]

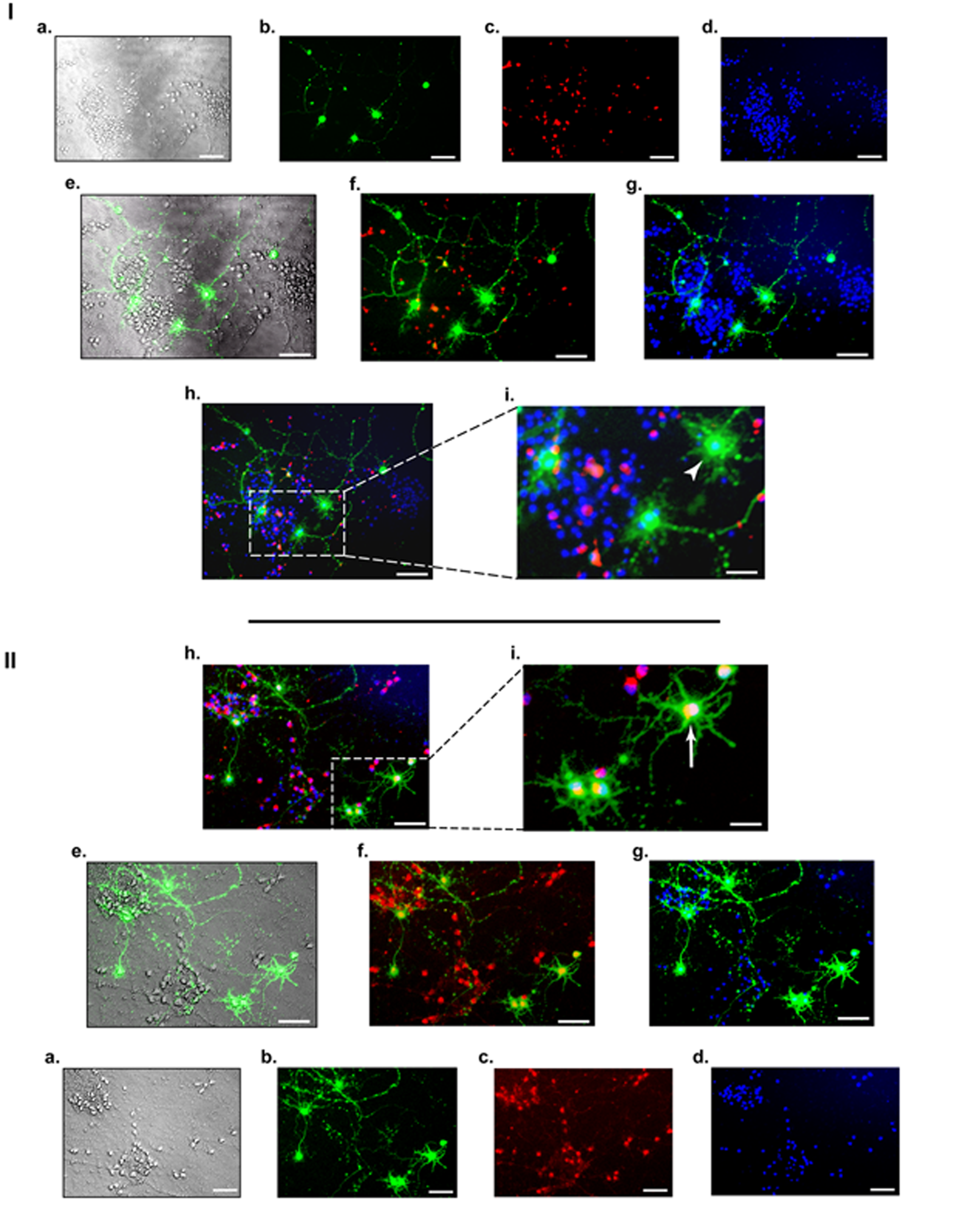

Supplement: Figure S4 — Over-expressed ZEB1 mitigates cellular damage/death in primary cortical neurons subjected to a battery of toxic insults: effect on mitochondrial membrane integrity. Primary cultures of cortical neurons were co-transfected with either GFP alone (White Bars) or a full-length cDNA for ZEB1 fused to GFP (Blue Bars). Eighteen hours later cells were subjected to a battery of pro-death/toxic insults (for details, see Materials and Methods). GFP-positive neurons were processed and scored (in a blinded fashion) for either intact (rhodamine stain) or compromised (absence of rhodamine stain) mitochondrial membranes. In this example, representative photomicrographs of cells challenged with OGD for 6 hrs indicate that a greater percentage of ZEB1-transfected cells maintain mitochondrial integrity, than those transfected with GFP alone (panels h and i). For every administered insult (except nitric oxide- see text) in an acute time-frame, and in a dose-dependant manner, mitochondrial integrity in ZEB1-transfected neurons was maintained relative to neurons transfected with GFP alone. Results for six different insults are summarized graphically in Figure 3. In both panels, a, phase contrast; b. GFP-positive cells; c. TMRhodamine-red-positive cells, indicating intact mitochondria; d. Hoechst staining of nuclei; e. phase plus GFP; f. GFP plus TMR-red; g. GFP plus Hoechst; h. triple stain; i. magnification of indicated area in panel h. Scale bars, a–h = 50 µm; I = 20 µm. (4.08 MB TIF) [file pone.0004373.s004.tif]

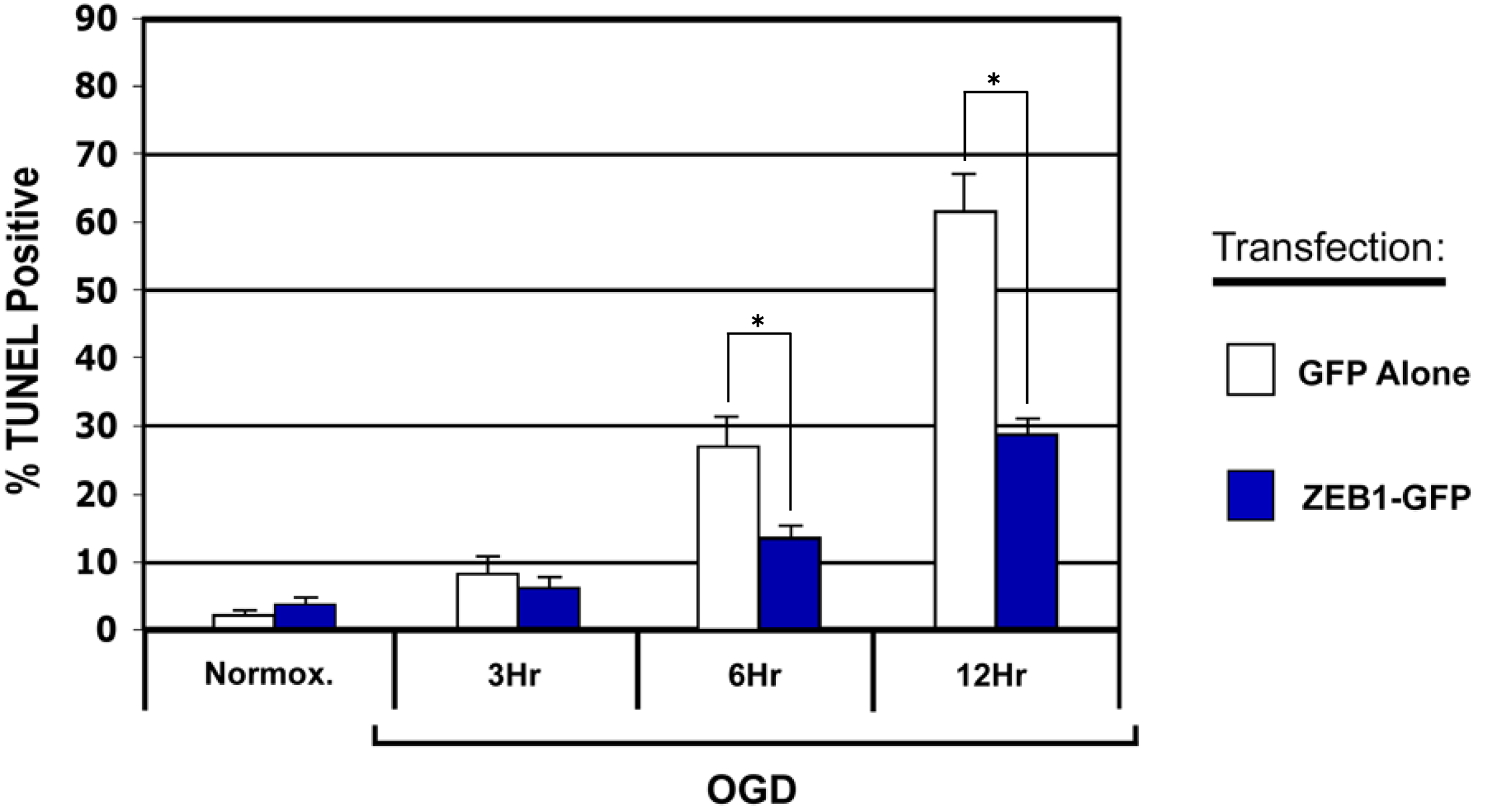

Supplement: Figure S5 — Over-expressed ZEB1 protects primary cortical neurons from OGD-mediated cellular damage/death: time course of TUNEL-labeling. Primary cultures of cortical neurons were co-transfected with either GFP alone (White Bars) or a full-length cDNA for ZEB1 fused to GFP (Blue Bars). Eighteen hours later, cells were subjected to OGD for the times indicated, fixed under hypoxic conditions, and GFP-positive cells were scored for TUNEL staining in a blinded fashion. Consistent with the results from Figure 3, over-expressed ZEB1 reduced the average number of TUNEL-positive cells by less than half at the 12 hr time point. Results shown are the average of three separate experiments (cultures isolated on different days)+/−the S.E.M.; * = P<0.01 by Student's T-test. (9.48 MB TIF) [file pone.0004373.s005.tif]

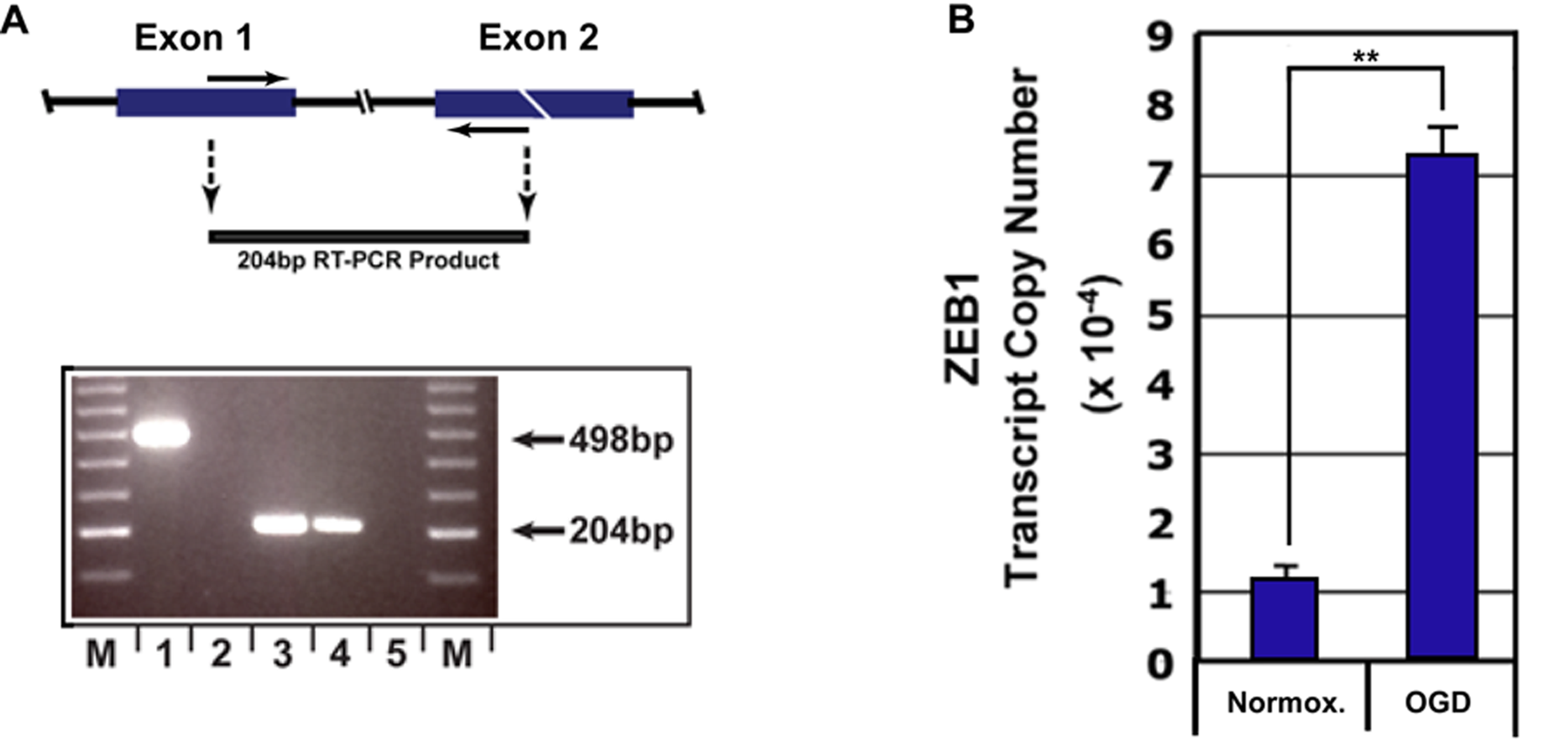

Supplement: Figure S6 — Oxygen-glucose deprivation increases steady-state levels of ZEB1 mRNA in primary neuronal cultures. (A) Top Panel, Primers used in this analysis (see Materials and Methods for sequences) cross intron 1 of the mouse ZEB1 gene to yield a 204 bp PCR product. Bottom Panel, Control PCR reactions demonstrating ZEB1 primer specificity. Lane 1, 498 bp SIP1 PCR product derived using SIP1-specific primers and SIP1 cDNA template; lane 2, 1 µg SIP1 cDNA template with ZEB1-specific primers; lane 3, 204 bp product derived using ZEB1-specific primers with 1fg ZEB1 cDNA template; lane 4, 204 bp RT-PCR product using ZEB1-specific primers and 10 ng total RNA isolated from an E16.5 rat primary cortical culture; lane 5, same as 4 minus RT; M, 100 bp incremental markers. (B) Quantitative RT-PCR results using indicated ZEB1-specific primers and 10 ng total RNA isolated from E16.5-derived primary cortical cultures subjected either to normoxia or OGD (3 hrs. with RNA harvested under hypoxia). Values are numbers of transcripts of ZEB1 mRNA (derived from a standard curve generated concurrently in the same PCR run) per µg of input total RNA. (0.45 MB TIF) [file pone.0004373.s006.tif]
